# Genetic basis and detection of unintended effects in genetically modified crop plants

**DOI:** 10.1007/s11248-015-9867-7

**Published:** 2015-02-26

**Authors:** Gregory S. Ladics, Andrew Bartholomaeus, Phil Bregitzer, Nancy G. Doerrer, Alan Gray, Thomas Holzhauser, Mark Jordan, Paul Keese, Esther Kok, Phil Macdonald, Wayne Parrott, Laura Privalle, Alan Raybould, Seung Yon Rhee, Elena Rice, Jörg Romeis, Justin Vaughn, Jean-Michel Wal, Kevin Glenn

**Affiliations:** 1 DuPont Pioneer Agricultural Biotechnology, DuPont Experimental Station, 200 Powder Mill Road, Wilmington, DE 19803 USA; 2 Therapeutics Research Centre, School of Medicine, Queensland University, Brisbane, QLD 4072 Australia; 3 Faculty of Health, School of Pharmacy, University of Canberra, Locked Bag 1, Canberra, ACT 2601 Australia; 4 National Small Grains Germplasm Research Facility, US Department of Agriculture – Agricultural Research Service, 1691 S. 2700 W., Aberdeen, ID 83210 USA; 5 ILSI Health and Environmental Sciences Institute, 1156 15th St., NW, Suite 200, Washington, DC 20005 USA; 6 Centre for Ecology and Hydrology, CEH Wallingford, Crowmarsh Gifford, Wallingford, Oxfordshire OX10 8BB UK; 7 Division of Allergology, Paul-Ehrlich-Institut, Paul-Ehrlich-Strasse 51-59, 63225 Langen, Germany; 8 Cereal Research Centre, Agriculture and Agri-Food Canada, 101 Route 100, Morden, MB R6M 1Y5 Canada; 9 Office of the Gene Technology Regulator, Australian Government, MDP54, GPO Box 9848, Canberra, ACT 2601 Australia; 10 RIKILT Wageningen UR, P.O. Box 230, 6700 AE Wageningen, The Netherlands; 11 Canadian Food Inspection Agency, 1400 Merivale Rd, Ottawa, ON K1A 0Y9 Canada; 12 Center for Applied Genetic Technologies, University of Georgia, 111 Riverbend Road, Athens, GA 30602 USA; 13 Bayer CropScience, 407 Davis Drive, Morrisville, NC 27560 USA; 14 Syngenta Ltd, Jealott’s Hill International Research Centre, Bracknell, RG42 6EY UK; 15 Department of Plant Biology, Carnegie Institution for Science, 260 Panama St., Stanford, CA 94305 USA; 16 Monsanto Company, 700 Chesterfield Pkwy W., CC5A, Chesterfield, MO 63017 USA; 17 Agroscope, Institute for Sustainability Sciences ISS, Reckenholzstr. 191, 8046 Zurich, Switzerland; 18 University of Georgia, 111 Riverbend Road, Athens, GA 30602 USA; 19 Dept. SVS, AgroParisTech, 16 rue Claude Bernard, 75231 Paris, France; 20 Monsanto Company, 800 N. Lindbergh Blvd, U4NA, St. Louis, MO 63167 USA; 21 Present Address: Syngenta Crop Protection AG, Schwarzwaldallee 215, 4058 Basel, Switzerland

**Keywords:** Unintended effects, GM crop plants, Environmental risk assessment, Allergenicity, Toxicity

## Abstract

In January 2014, an international meeting sponsored by the International Life Sciences Institute/Health and Environmental Sciences Institute and the Canadian Food Inspection Agency titled “Genetic Basis of Unintended Effects in Modified Plants” was held in Ottawa, Canada, bringing together over 75 scientists from academia, government, and the agro-biotech industry. The objectives of the meeting were to explore current knowledge and identify areas requiring further study on unintended effects in plants and to discuss how this information can inform and improve genetically modified (GM) crop risk assessments. The meeting featured presentations on the molecular basis of plant genome variability in general, unintended changes at the molecular and phenotypic levels, and the development and use of hypothesis-driven evaluations of unintended effects in assessing conventional and GM crops. The development and role of emerging “omics” technologies in the assessment of unintended effects was also discussed. Several themes recurred in a number of talks; for example, a common observation was that no system for genetic modification, including conventional methods of plant breeding, is without unintended effects. Another common observation was that “unintended” does not necessarily mean “harmful”. This paper summarizes key points from the information presented at the meeting to provide readers with current viewpoints on these topics.

**Electronic supplementary material:**

The online version of this article (doi:10.1007/s11248-015-9867-7) contains supplementary material, which is available to authorized users.

## Introduction

As genetically modified (GM) crops worthy of commercialization became available, procedures were instituted to ensure that these plants were as safe for food, feed, and environmental release as their conventional (non-GM) counterparts. These procedures addressed two broad categories of changes that could be considered in a GM crop safety assessment: intended and unintended. The intended change in a new GM product is the desired phenotype brought about by the introduced transgene. Because many transgenes express a known and characterized protein, procedures can be developed that directly assess the protein for toxicity and allergenicity and measure levels of metabolites that may be associated with the protein’s function. Unintended changes, on the other hand, could materialize as a consequence of gene insertion, from random mutations that take place during the transformation and tissue culture process, or from pleiotropic effects of the introduced protein, and there is no single direct test for them.

In January 2014, an international meeting sponsored by the International Life Sciences Institute/Health and Environmental Sciences Institute and the Canadian Food Inspection Agency titled “Genetic Basis of Unintended Effects in Modified Plants” was held in Ottawa, Canada, bringing together over 75 scientists from academia, government, and the agro-biotech industry. The objectives of the meeting were to explore current knowledge and identify areas requiring further study on unintended effects in plants and to discuss how this information can inform and improve GM crop risk assessments.

The potential for an unintended effect to present a food or feed hazard is currently assessed through compositional analyses and agronomic studies to compare the GM crop with a genetically similar conventional counterpart. Some regulatory authorities, such as those in the European Union (EC 2013), may also require animal feeding tests. Some aspects of testing for unintended effects seem to be generally accepted, such as the use of related conventional comparators. Nevertheless, many questions are still being discussed, for example, whether it is sufficient to limit testing for unintended effects to those subject to testable hypotheses, or whether (and when) the precautionary principle requires a broader look for genomic changes via profiling methods. As noted during the opening presentation and by several other speakers, “unintended” is not synonymous with “harmful” (e.g., NRC 2004).

This manuscript summarizes four broad areas discussed at the meeting: the molecular changes associated with plant genetic variability, the types of unintended genomic changes in GM plants, the use of hypothesis-driven evaluations of unintended effects, and the use of emerging technologies in the assessment of unintended effects. This paper is based on the meeting presentations, with new and updated information added where appropriate. For each section, the primary contributors are noted, but comments and edits from other authors have been included. The authors’ individual papers in their entirety are available as Online Resource 1. Presentations and other information from the meeting can be found at http://www.hesiglobal.org/i4a/pages/index.cfm?pageID=3654.

## Molecular basis of plant genetic variability^1^

High-throughput sequencing and other genomic technologies have made it possible to evaluate the nature and extent of naturally occurring genomic changes in plants. These were extensively reviewed by Weber et al. (2012), who noted the following:

- Single-nucleotide changes are common, with a background rate of seven new mutations per billion bp of DNA, or roughly seven new mutations for every soybean (*Glycine max*) plant in every field (Ossowski et al. 2010).
- Insertions from transposons can be very common as well, with rates as high as 50 novel insertions per plant per generation reported in a variety of rice (*Oryza sativa*; Naito et al. 2006). Transposon insertions are also very common in soybean (Tian et al. 2012).
- Plants create novel genes through transposon capture, whereby pieces of different genes are assembled in novel combinations (reviewed in Weber et al. 2012).
- There are genes that are present in different numbers or absent altogether in different individuals within a crop (e.g., Lai et al. 2010; Lam et al. 2010; Potato Genome Consortium 2011; McHale et al. 2012).
- Horizontal gene transfer is not uncommon, with pararetroviral (double-stranded DNA virus) sequences being particularly abundant in the genomes of crop plants (e.g., Liu et al. 2012; Staginnus et al. 2007).

The effects of naturally occurring insertions are of particular interest because plant genetic engineering is typically mediated by the insertion of a modified T-DNA sequence from *Agrobacterium tumefaciens* or other vector DNA sequences into the genome. This insertion may potentially disrupt the function of native genes and can create rearrangements at the site of insertion. Indeed, roughly half of T-DNA insertions exhibit less than 8 bp of “filler” DNA at the junction site, while the other half contain larger additions, generally between 8 and 100 bp (Forsbach et al. 2003). Short insertions, comparable to those seen at T-DNA junctions, have been observed in induced double-strand break experiments (Lloyd et al. 2012; Vu et al. 2014). Such insertion variation is common, even in closely related rice varieties, and reflects the fact that errors in double-strand break repair are frequent in natural and breeding populations (Vaughn and Bennetzen 2014). Thus, the “filler” DNA observed in T-DNA insertions has a clear counterpart in naturally occurring DNA insertions.

In summary, the view that only transgenes result in insertions, and that these have a unique ability to disrupt gene expression (e.g., Fagan et al. 2014) is not supported by the available data. Instead, plant genomes are very dynamic and plastic, as predicted by Barbara McClintock (1984) in her Nobel address, and undergo frequent insertions and other rearrangements.

## Molecular basis of unintended effects in GM plants

In addition to the potential for insertional effects of transgenes, other mechanisms such as somaclonal variation and pleiotropy can contribute to unintended effects.

### Somaclonal variation^2^

Semi-differentiated plant tissues cultured in vitro are critical for most plant transformation methods. In vitro culture induces genetic and epigenetic changes, termed somaclonal variation (SCV; Larkin and Scowcroft 1981), that are another possible source of unintended variation. Although SCV is potentially useful as a source of novel mutations, it is contrary to the objective of making limited, predictable changes as a result of transgene introduction. For example, significant and negative changes have been noted in the agronomic performance and malting quality of tissue-culture-derived barley. Yield losses of 15–84 % have been observed in non-transgenic derivatives of transgenic plants, i.e., non-transgenic segregants derived from heterozygous transgenic plants (Bregitzer et al. 1998). Although certain adjustments to the in vitro environment can reduce the severity of SCV, the most effective way to eliminate it in barley has been to backcross transgenic plants to plants without any SCV (such as the wild-type parent used in making the original transgenic plant), with selection at each generation based only on the presence of the transgene. For example, a single backcross to barley cultivar Conlon recovered the majority of yield loss caused by SCV in a group of transgenic Conlon-derived lines. On average, the yield loss in the primary transgenic lines was 31 %, compared with 6 % in the backcross-derived lines (Bregitzer and Dahleen 2008).

### Pleiotropic effects of transgenes^3^

Some unintended effects might be caused by pleiotropy, the effect of a single gene (whether a native gene or a transgene) on multiple traits (Fagan et al. 2014). When both positive and negative effects are caused by the same gene, it is referred to as antagonistic pleiotropy. An example of antagonistic pleiotropy is the wheat (*Triticum aestivum*) gene *Lr34*, which encodes an ABC transporter, a molecule involved in the transport of metabolites across membranes (Krattinger et al. 2009). *Lr34* provides durable resistance to a number of wheat diseases; however, it also causes premature senescence of the flag leaf (leaf tip necrosis) that can reduce potential yield in the absence of disease. If the wheat *Lr34* gene is moved into barley (*Hordeum vulgare*), the negative effect becomes stronger and the plants exhibit stunted growth and sterility (Risk et al. 2013). One possible explanation is that wheat has regulatory mechanisms that control the expression of the gene in a manner that minimizes its negative effects; on the other hand, the barley lines tested were primary transgenic lines and had not undergone selection against SCV (described in the previous section). The amount of leaf tip necrosis varies among wheat genotypes, but plant breeders have selected lines that maximize the benefit while reducing the negative effect of the gene. The same may be possible in barley if the detrimental effects seen are due to somaclonal variation rather than to pleiotropy.

Prediction of whether pleiotropy (and therefore the possibility of unintended effects) is likely to occur as the result of a transgene depends on knowledge of the biochemical mechanism of the encoded protein. Genes affecting basic cellular functions that are needed by many traits (such as ABC transporters) are more likely to be pleiotropic. Similarly, genes in which alternative splicing occurs in the pre-mRNA or that encode a protein affecting multiple pathways (e.g., transcription factors or other regulatory proteins or molecules) could potentially be pleiotropic.

A gene’s origin may also be an indicator of whether pleiotropy is likely to occur, but this is harder to predict. There could be more pleiotropy if the gene originates from another species due to lack of regulatory controls (e.g., expression of wheat *Lr34* in barley) or less pleiotropy due to lack of a pathway or function in the recipient species compared with the donor (original) species. An example of this is the lack of anthocyanin production in transgenic tomato (*Lycopersicon esculentum*) fruit after the introduction of genes regulating flavonol and anthocyanin production from maize (*Zea mays*) (Bovy et al. 2002). The tomato plants had increases in some flavonols but no anthocyanin production because tomato lacks sufficient expression of another gene required for anthocyanin production. Variation in regulatory mechanisms is not only observed at the species level—different genotypes of the same species can have variation in regulatory mechanisms (e.g., Schiessl et al. 2014), which is one reason there is so much phenotypic and phenologic diversity in crop species. Without this type of diversity, selection during plant breeding would be less effective.

### Similarity between unintended effects in conventional plant breeding and biotechnology^4^

Plant breeders have successfully improved crop yields despite having little or no information on the genes and gene networks that impact yield. In the case of maize, it is clear that improvements in grain yield have been associated with significant changes in many other traits (Tollenaar and Lee 2010). Whereas in conventional plant breeding, the exact functions of the combined genes are mainly unknown, biotechnology enables the introduction of specific genes with expected effects on endogenous pathways and phenotypes.

Although crop plants derived from conventional plant breeding and GM differ in the level of molecular data required for commercialization, both conventional breeding products and GM products undergo a similar process of selection for intended characteristics and elimination of undesirable phenotypes (Privalle et al. 2012). While it is clear that unintended effects occur in any type of breeding program, including conventional crossing (Cellini et al. 2004; Kok et al. 2008; Schnell et al. 2015), the discussion on the potential for unintended events tends to be focused on GM organisms.

This similarity between the changes caused by biotechnology and conventional plant breeding is reflected in the approach to regulation used in Canada. The Canadian regulatory scope covers plants developed to possess characteristics sufficiently different from those of the same or similar species, regardless of the method used. As a consequence of the product-based regulatory approach, in Canada the regulated plant is referred to as a plant with a novel trait (PNT). PNTs include GM crops as well as some produced by more conventional breeding techniques. The approach is designed to take advantage of the knowledge, expertise, and regulatory framework that are already present in regulatory departments and agencies but applied to conventional products. It is also an acknowledgment that the Canadian Government policy considers that PNT crops should be considered as an extension of conventional breeding techniques and that the focus of assessment should be on the novel trait rather than on how the trait was obtained.

### The GM product development process^5^

The extensive vetting involved in the generation and selection of one or a few “elite” (i.e., top-performing) events minimizes the likelihood of unsafe unintended effects associated with the GM crop products that are taken to commercialization. Many ideas, traits, and events are evaluated to identify an event for which it is worth seeking approval (e.g., Phillips McDougall 2011; Privalle et al. 2012). As noted earlier, this is not unlike the approach taken by breeders seeking to develop a new and improved variety.

For GM crops, as for those derived from conventional breeding, the most important selection criterion is efficacy/performance (i.e., does the trait impart the desired phenotype, meeting product specifications). In the case of GM crops, the next most important criteria applied in identifying the lead event are those related to the molecular characteristics of the event. The inserted DNA ideally should be present at a single locus and as a single copy. There should be no vector backbone present in the event and the insertion should not have disrupted an endogenous gene or created a chimeric novel fusion protein. There should be minimal locus rearrangement and the integrity of the gene cassette should have been preserved. Importantly, while none of these parameters have been demonstrated to impact the safety of the crop, the consideration of these parameters is based on hypothetical, minimal-probability possibilities. Since most GM products require multiple approvals, the requirements from the strictest jurisdictions dominate the event selection criteria.

Once the elite event is identified, an extensive safety assessment is conducted that includes studies on the safety of the newly expressed protein, molecular characterization of the insert, impact of the insert on plant performance and composition, environmental impact, and wholesomeness of the crop (SCBD 2000; Codex 2003). The assessment includes phenotypic and agronomic comparison between the new plant variety and a genetically similar comparator that is already on the market and considered as safe. GM foods are among the most highly studied foods consumed, and the registration dossiers are scrutinized by regulatory agencies around the world. To date, approval has not been withheld for any event based on an unintended effect.

## Hypothesis-driven evaluation of unintended effects

### Approaches to risk assessment^6^

Risk is a combination of the seriousness and likelihood of a harmful effect following a course of action. Risk assessment characterizes the amount of risk associated with an activity. It contributes to making decisions about whether to undertake an activity, such as the import, field testing, or cultivation of a specific GM crop. Some authors have raised concern (e.g., Craig et al. 2008) that the amount of data required for risk assessments of GM crops is increasing and becoming detrimental to decision-making in many countries.

Two possible approaches to risk assessment have been described. In the “bucket” approach (Raybould 2011), data on the properties of the GM crop are collected in an untargeted manner, often termed profiling. Profiling could comprise measurements of the crop’s gross phenotype, composition of key tissues, transcriptome, proteome, metabolome, and so on. By comparing these profiles with those of a suitable conventional crop, the risk assessor is supposed to be able to identify changes, which in turn indicate that there may be changes in the GM crop that are potentially harmful (see “Omics technologies” below).

There are several limitations to this approach. First, what to regard as harmful is defined by policy; it is not discovered in data (Sarevitz 2004; Sanvido et al. 2012). Second, even if harm is defined, profiling will collect data that do not predict the seriousness or probability of harm following use of the GM crop. These data are thus irrelevant for risk assessment and may impair decision-making because they distract from data that are relevant. Furthermore, it is difficult to interpret the effect of an altered metabolite in the absence of a hypothesis.

The second approach regards risk assessment as a hypothesis-testing exercise. The risk assessor identifies those effects that would be regarded as harmful if they were to occur, based on relevant legislation or regulations (Evans et al. 2006), and builds scenarios comprising a series of events leading from the proposed use of the particular GM crop to the identified harmful effects. These scenarios, or “pathways to harm”, allow the risk assessor to devise testable hypotheses about the likelihood, frequency, or magnitude of the events in the pathway. Data are collected to test these hypotheses and thereby characterize risk (Raybould 2011).

A concern raised about the latter approach is that it represents a biased approach to assessment. Effective risk assessment does involve bias in that representative protection goals must be selected from among all the possible effects of using a GM crop. Limited resources are then targeted to test hypotheses about the probability and consequences of those effects. These will be strong tests of clear hypotheses, which, if corroborated, provide high confidence in conclusions of low risk.

### Problem formulation in environmental risk assessment^7^

Environmental risk assessment (ERA) for GM crops deals almost exclusively with the phenotype and considers all plant traits that may have been altered by the transformation, whether intended or unintended. Of particular interest are any unintended changes in traits that may make the GM plant more persistent or invasive (“weedy”) in either agricultural or natural environments. These include changes in the properties of the seeds (such as developmental rates, number, release from the plant [shattering], dormancy, and germination rates) that are important in the “regeneration niche” of the plant’s establishment and spread, and in those traits that affect the plant’s competitiveness (such as seedling vigor, plant height, growth rates, and resistance to pests and disease).

The first stage in problem formulation in an ERA is to identify a set of environmental protection goals derived from local, national, or international policy. These may be broadly stated (e.g., the Cartagena Protocol) or more specific laws, statutes, or even guidelines, but collectively they enable a risk assessor to identify those aspects of the environment that must be protected. These can sometimes be formally defined in terms of assessment endpoints (e.g., “insect pollinator abundance”), which are whatever will be measured to ascertain whether protection has been achieved as intended (Paes de Andrade et al. 2014).

The second stage of problem formulation is to seek a link between the cultivation of the GM crop and the assessment endpoint that may result in harm (i.e., the pathway to harm). For example, insect pollinators are likely to be harmed if the plant presents a hazard (e.g., an insecticidal protein that negatively affects the insect) to which the insect may be exposed. Steps along the pathway to harm can be recast as risk hypotheses that can be validated or rejected from existing data, or by designing new experiments or trials where appropriate. For example, validation of the hypothesis “the insect is not harmed by the protein” or “the protein is not expressed in pollen” allows a confident risk assessment without further experimentation. Wolt et al. (2010) describe the process in detail and Raybould (2011) and earlier papers referred to therein give specific examples of formulating and testing risk hypotheses. Gray (2012) and Tepfer et al. (2013) give practical examples of the use of problem formulation in ERA for GM crops.

### Evaluation of food and feed safety

As mentioned earlier, the approach to food and feed safety described by the Codex Alimentarius Commission (Codex 2003) is based on comparison of the GM crop to a conventional counterpart employing a weight-of-evidence approach. In this way, the assessment is focused on identification of potential new hazards or changes in hazard levels in the GM product.

#### Focus on plausible outcomes^8^

For food and feed safety risk assessment of GM crops, it is necessary to exclude hypothetical, extreme, or scientifically implausible circumstances. Instead, the purpose is to (1) identify practical, biologically plausible outcomes based on the extensive data now available, and (2) to define the nature of the at-risk group(s) to be addressed (population or individual health risks), the type of hazard(s) of concern (toxicological, dietary, immunological), and the risk time metric (acute, sub-chronic, or chronic).

It is unlikely that systemically toxic proteins that are unrelated to the parent plant variety or to the function of the transgene will be produced de novo in a GM plant (Weber et al. 2012). The reason is that systemic toxicity of an ingested protein requires at least three highly specific structural characteristics: (1) resistance to digestion, (2) ligand specificity for the gut uptake transporters, and (3) ligand/receptor specificity for site- and species-specific receptor-mediated toxicity (Hammond et al. 2013). A change in any one of these three characteristics is an implausible outcome from either conventional plant breeding or gene transfer; thus, the probability of having all three occur in the same plant is vanishingly small. Similarly, the potential for random genome effects to modify existing non-toxic proteins to create a toxic protein is also essentially zero (Weber et al. 2012). This prediction is evidenced by the current knowledge of conventional corn and other food crop varieties that have millions of single-nucleotide polymorphisms (SNPs) across genotypes (Tenaillon et al. 2001), but have never resulted in a novel toxic protein in a food crop (Steiner et al. 2013).

The de novo generation of the machinery necessary to produce a toxic secondary metabolite is also very unlikely. Such an event has not been observed in the extensive range of varieties produced by genetic manipulation in conventional and GM crop breeding over the past century. The reactivation of dormant pathways (i.e., pathways present in an ancestor of the crop that are inactive in the modern variety) has also never been observed and is implausible due to the accumulation of mutations in non-functional DNA, progressively degrading any residual potential functionality (Steiner et al. 2013).

As in conventional plant breeding, there are natural variations in the levels of compounds, including those of toxicological relevance, in crops developed through modern biotechnology. Examples of plausible mechanisms include the up (and down) regulation of pre-existing endogenous plant toxins, increased/decreased uptake of heavy metals from the soil or water (e.g., Cd, As, Se), altered levels of nutrients or antinutrients associated with population health outcomes, altered production of pesticide metabolites, altered levels of toxic substrates (precursors) due to blocking of an enzyme pathway, and altered release or availability of endogenous toxins.

#### Assessment for changes in levels of existing allergens^9^

According to the European Union perspective on the assessment of the overall allergenicity of whole GM plants, in cases where the recipient species of a genetic modification is a known allergen, (e.g., soybean) the qualitative and quantitative composition of endogenous allergens in the GM crop and its conventional counterpart should be compared (Metcalfe et al. 1996). The concentration of endogenous allergens within a plant species is highly variable, and the comparative analysis should consider the influence of the cultivars and of the conditions of cultivation, harvest, storage, and processing on the expression of allergens (see “Examples of natural variation in allergens” below). The European Food Safety Authority (EFSA) guidance and the European Commission (EC) regulation have recommended including “key” endogenous allergens (i.e., such as those listed in the OECD consensus documents) in the comparative compositional analysis of plant materials collected from controlled field trials. This aims to assess whether the GM plant is more allergenic than its conventional counterpart (EFSA 2011; EC 2013). As described by the EC, “key allergens” are well-characterized allergens that are relevant for public health because of their allergenic potency and abundance. They are generally well-conserved proteins with important metabolic and physiological functions in the plant, such as enzymes, defense proteins, or storage proteins. Any significant change in key allergen levels could thus be directly related to the specific allergy risk and could also indicate the possible occurrence of other types of unintended effects.

Non-targeted analyses, such as proteomic approaches using mass spectrometry (e.g., matrix-assisted laser desorption/ionization (MALDI) or electrospray ionization time-of-flight mass spectrometry (ESI-TOF MS) in combination with different separation methods such as 2-dimensional gel electrophoresis or liquid chromatography), have been rapidly developed (Goodman et al. 2013) and can help to identify significant changes in endogenous allergen expression. They may not require human sera and have proven to be efficient (alternative) tools for the identification and quantification of known allergens in plants. However, such tests may sometimes be considered as complex and insufficiently standardized and needing further developments and validation before they can be routinely used for safety assessment (Fernandez et al. 2013).

#### Examples of natural variation in allergens^10^

Allergies to fruits and vegetables affect up to ~4 % of the population in Europe (Zuidmeer et al. 2008). Carrot (*Daucus carota*) and apple (*Malus domestica*) are among the most prevalent elicitors of allergic reactions to foods in northern and central Europe. In apple, variation in patient reaction and/or allergen levels has been observed among cultivars (Bolhaar et al. 2005), stored vs. unstored fruit (Sancho et al. 2006a, b), and patient geographical areas. Similarly, the carrot isoallergens (related allergens from the same species) Dau c 1.01 and Dau c 1.02 were quantified using ELISA (Foetisch et al. 2011) in two cultivars, ‘Rodelica’ and ‘Nerac’, in a two-year study. Initial evaluation of the field data suggests a large influence of the year of cultivation and an apparent difference between the two cultivars (unpublished data, research project BÖL 03OE349 granted by the German Federal Ministry of Food, Agriculture and Consumer Protection). Furthermore, some isoallergens might be more relevant than others for clinical reactivity, and the level of allergens can increase or decrease depending on genetic and environmental factors. Studies on the allergenicity of apple and carrot have focused on non-transgenic cultivars; however, they can be considered model foods to study the influence of genetic and environmental factors on the composition of panallergenic structures (functionally related allergenic molecules found in different species) and the isoallergen distribution in fruits and vegetables. Understanding the biochemical pathways of allergen synthesis and the range of natural variability may support hypothesis-driven studies on unintended effects in GM plants intended for human consumption.

### Environmental risk assessment of GM crop plants

#### Example of hypothesis-driven testing: drought-tolerant maize^11^

The evaluation of Monsanto’s recently introduced DroughtGard^®^ maize hybrids (event MON 87460) was used as a case study to illustrate how hypothesis-driven testing can be used for safety assessment. This product expresses a bacterial cold shock domain protein B (*Bacillus subtilis* CSPB), which imparts reduced yield loss under water-limited conditions compared with conventional corn (Castiglioni et al. 2008). CSPB is a member of the cold shock domain-containing (CSD-containing) protein family. Under environmental stress, CSD-containing proteins moderate stress responses in bacteria and plants, primarily through stabilization of RNA and improved cellular function (Cristofari and Darlix 2002; Chaikam and Karlson 2008). Like endogenous CSD proteins found in bacteria and plants, the CSPB protein in MON 87460 interacts with RNA and accumulates and localizes to rapidly growing tissues and in developing reproductive organs, thereby helping to maintain cellular function in those tissues during stress (Nemali et al. 2014). Under water-limited conditions, there is a trend toward improved ear growth rate for MON 87460 compared with the control plants, while the common mechanisms of plant response to drought stress are not altered in transgenic CSPB-expressing maize plants (Castiglioni et al. 2008; Nemali et al. 2014). When plants were grown under well-watered conditions, no appreciable differences between CSPB-expressing lines and the control were detected (Castiglioni et al. 2008).

Based on the understanding of the CSPB mode of action, the ERA for MON 87460 included six hypothesis-driven studies that answered specific questions relevant to the nature of the trait, in addition to the standard phenotypic and agronomic field trials in the presence and absence of the trait (Sammons et al. 2014). The studies included assessments for persistence outside of cultivation; root growth and development; and drought, cold, heat, and salt tolerance (Sammons et al. 2014). No additional abiotic stress tolerances were identified and no differences in season-long water consumption or root growth and development were observed. These studies did not reveal any potential for adverse environmental impacts.

#### Example of hypothesis-driven testing: assessment of potential for weediness^12^

In Australia, previous experience on assessment of weediness has been used to assess transgenic crops. Identification of characteristics that are relevant to weediness/invasiveness has been based on practical experience with more than 1200 major environmental and agricultural weeds in diverse landscapes (Randall 2012). Weed scientists have produced a robust and simple weed risk assessment protocol that can be readily applied to any plant (Keese et al. 2014). In addition, the large datasets available from weed risk assessments include plants across the whole risk spectrum and allow rigorous validation tests to be conducted (Virtue et al. 2008; Stone and Byrne 2011).

The most advanced method for weed risk assessment is based on the post-border weed risk management protocol (Auld 2012), which was developed as a means of prioritizing existing weeds for control. It can be adapted to risk assessment of GM plants (Keese et al. 2014) by comparing the weed risk of the GM plant to that of the conventional counterpart. This approach is used to identify significant changes based on three factors: the risk context (i.e., the environment where the GM crop might be present), the ability of the GM plant to spread and persist, and the potential negative impacts on biodiversity, non-target organisms, soil nutrients, etc.

The weed risk approach specifies relevant characteristics of GM plants that affect spread and persistence (invasiveness) and those that potentially give rise to negative impacts on human or animal health, or the environment. These characteristics capture changes due to either intended or unintended effects. Changes that have no or negligible effect on weed risk need not be explored. The post-border weed risk assessment approach therefore provides guidance on the data requirements, for both intended and unintended traits, that are considered relevant for the ERA of a GM plant.

#### Unintended effects on non-target organisms^13^

A common concern associated with the growing of GM crops is over their potential to have adverse impacts on non-target organisms. Arthropods in particular form a major part of the biodiversity in agricultural landscapes, and many are valued because they provide important ecosystem services, including biological control, pollination, and decomposition, or cultural services, including human enjoyment and education (Sanvido et al. 2012; Garcia-Alonso and Raybould 2014). Therefore, potential impacts that GM plants may have on valued non-target arthropods (NTAs) are addressed in ERA.

Both the intended change (e.g., production of a Bt Cry [crystal] protein for target insect control) and any unintended changes could cause unintended effects on valued non-target organisms. Since consequences of the intended change can be anticipated, it is possible to construct conceptual models (pathways to harm) of how growing of the GM plant could harm valued NTAs and to formulate risk hypotheses that can subsequently be tested (Raybould 2011). A common hypothesis is that the stressor (i.e., the Cry toxin) does not reduce the abundance and ecological functions of NTAs under field conditions. This hypothesis is typically tested within a tiered framework that moves from laboratory or early-tier tests using species that are readily available, amenable to testing, and able to detect potential hazards, to more complex (higher-tier) experiments that evaluate the risks under more realistic exposure conditions such as field studies (Romeis et al. 2008). Laboratory studies (termed tier 1 tests) are particularly powerful for testing the risk hypothesis; in cases where no adverse effects are detected under these highly controlled laboratory and worst-case exposure conditions, a “no effect” conclusion can be drawn with high confidence (Raybould et al. 2007; Romeis et al. 2011).

In the case of unintended, plant-transformation-related effects, the assessment typically follows a weight-of-evidence approach taking into account information from the molecular characterization of the particular GM event and from comparisons of composition and agronomic and phenotypic characteristics of the GM plant with its conventional counterpart (Garcia-Alonso and Raybould 2014). If differences are detected, their likely biological relevance will be assessed by considering the range of values known for the conventional crop varieties that have a history of safe use. The aim of this assessment is to identify potentially harmful unintended changes that, if found, would trigger a more detailed assessment (Romeis et al. 2008). This approach is considered sufficiently conservative given the fact that more than 99 % of all transformation events are eliminated during prior agronomic and phenotypic analyses (e.g., Phillips McDougall 2011).

It is sometimes argued that risk assessments should include experiments to study the impact of unintended, transformation-related effects on non-target organisms. For example, the EFSA requests non-target studies using GM plant material as a test substance to “… give indications on possible interactions between plant compounds and reflect realistic exposure conditions through bioavailability” (EFSA 2010). The justification for these additional data is that the compositional analyses do not necessarily target specific metabolites known to be involved in non-target-organism–plant relationships. This approach has many limitations. For example, it is usually unknown which metabolites are involved in these interactions, and different metabolites are likely to affect different non-target species differently. Furthermore it is more likely to detect differences in plant tissue composition among different plant varieties or even plant batches than between the tissue from GM plants and their non-transformed control (e.g., Meissle et al. 2014). Such experiments and their results may thus add confusion rather than certainty to the ERA. The published literature on the non-target impact of Bt maize, for example, provides a number of examples where studies using GM plant tissue as a test substance have resulted in inconclusive results (Romeis et al. 2013).

As a solution, the unintended, transformation-related effects that might adversely affect NTAs should be identified during the problem formulation phase of the ERA taking into account the results from the molecular, phenotypic, and agronomic characterization and the compositional analyses. If such characteristics are identified as stressors of concern, pathways to harm can be constructed and testable risk hypotheses can be formulated. This is a precondition to design and execute meaningful studies that provide data to support the ERA.

## Emerging technologies and application to assessment of unintended effects

### Omics technologies^14^

In contrast to the rationale described above suggesting that a hypothesis-driven approach is sufficiently informative for risk assessments, there are calls for the use of global profiling technologies to survey the plant more broadly than is currently feasible using a targeted approach. As previously noted, unintended effects can be found in both conventional and GM-based breeding programs (Kok et al. 2008; Weber et al. 2012; Flint-Garcia 2013). New plant breeding programs are moving toward a broader range of molecular biological breeding techniques to achieve more complex genetic alterations within the framework of shorter breeding programs (Lusser et al. 2011). In the future, other strategies that aim for even more profound changes, such as strategies based on synthetic biology, may be applied to plant breeding.

For these reasons, it is useful to have more informative and cost-efficient analytical methods to screen for unintended, potentially adverse, effects. Omics technologies may meet these criteria. Omics technologies, whether transcriptomics (mRNA profiling), proteomics (protein profiling) or metabolomics (metabolite profiling), have already shown their added value in different areas (Tanaka 2010; de Ligt et al. 2012; Rauch et al. 2012). In plant materials, these methodologies can be applied in a reproducible and informative way (Fernie and Schauer 2009; Van Dijk et al. 2010, 2012; Oms-Oliu et al. 2013). Such studies have confirmed that differences between a single-trait transgenic plant variety and its conventional counterpart will typically be smaller than differences between comparable conventional varieties for the analytes and time points examined.

New plant varieties could be screened for any aberrant omics profile, i.e., a profile that is different from the profiles of plant varieties considered as safe. New plant varieties for which compositional profiles fall within the range of profiles derived from plant varieties already considered safe would not require further assessment. This would not mean that a plant with an altered profile is not safe, but in the case of a profile outside the range of varieties considered as safe, a further detailed analysis of the new plant variety would be required to confirm its safety. This approach complements current approaches for targeted compositional analyses, but the information content of omics technologies will be much greater. Statistical and chemometric methods are available to rapidly screen profiles of new plant varieties with reference to profiles from plant varieties that we consider as safe (Van Dijk et al. 2014). To effectively implement omics technologies to improve current risk assessment procedures, it is necessary to establish simple, common protocols for omics analyses and related data analyses with the aim to (1) compare the new plant inbred or variety to an appropriate comparator (e.g., in the case of a GM crop, a conventional counterpart) and (2) compare the new plant inbred or variety to a larger set of genotypes of the same species that are considered safe. This approach will often relate to data that the plant breeder will already have available, thus considerably reducing the regulatory burden for plant breeders while safeguarding the food supply in the future.

### Genome-scale metabolic networks and metabolomics^15^

As suggested above, omics technologies have the potential for enabling comprehensive and quantitative assessment of metabolic changes in response to genetic modification. While non-targeted metabolite profiling can detect thousands of compounds, it is not easy to understand the significance of the changed metabolites in the biochemical and biological context of the organism. To derive biochemical explanations or hypotheses for the observed metabolite changes from non-targeted metabolomics studies, it is important to examine the changed metabolites in the context of a genome-scale metabolic network of the organism.

Much progress has been made in the last few decades to represent metabolism at a genome scale (Thiele and Palsson 2010). The advances in genome sequencing and emerging fields such as biocuration (biological database management) and bioinformatics enabled the reconstruction of genome-scale metabolic networks for model organisms (Bassel et al. 2012). Genome-scale metabolic networks have been predicted from reference metabolic pathway databases such as MetaCyc (Caspi et al. 2012), PlantCyc (Zhang et al. 2010), and KEGG (Kanehisa et al. 2012). These genome-scale metabolic networks are now available for several plant species such as *Arabidopsis thaliana* (Mueller et al. 2003; Zhang et al. 2010), poplar (*Populus trichocarpa*; Zhang et al. 2010), *Chlamydomonas reinhardtii* (May et al. 2009), medic (*Medicago truncatula*; Urbanczyk-Wochniak and Sumner 2007), grasses (Youens-Clark et al. 2011), and nightshade plants (Bombarely et al. 2011).

The genome-scale metabolic networks can be used to create predictive metabolic models (Sweetlove et al. 2008). Metabolic models can be used to predict metabolic fluxes under a variety of scenarios such as genetic perturbations (Feist and Palsson 2008). Two types of metabolic models have been used: kinetic and stoichiometric. Kinetic modeling uses enzyme kinetics to numerically simulate and test metabolic fluxes and can explain the mechanism of flux changes, but the difficulty of determining in vivo enzyme kinetics has limited this modeling to a small number of pathways. The other modeling approach uses the stoichiometry of reactants and products in reactions to solve for the most likely fluxes with added constraints based on thermodynamics, directionality, and flux capacity of reactions (Thiele and Palsson 2010). This approach has been used to build genome-scale metabolic models of several plant species such as Arabidopsis (Poolman et al. 2009; de Oliveira Dal’Molin et al. 2010), maize (Saha et al. 2011), and *C. reinhardtii* (Chang et al. 2011). Most of these models have not been validated extensively using flux measurements, though advances in metabolic flux analysis using ^13^C-labeling and metabolomics approaches hold promise (Schwender 2008; Sweetlove et al. 2008; Allen et al. 2009).

These metabolic models have been applied in a variety of studies ranging from metabolic engineering, drug discovery, drug target discovery, identification of novel gene function, evolutionary processes, network behaviors, and interpretations of mutant phenotypes (Feist and Palsson 2008). The most common algorithm used in these studies has been flux balance analysis (FBA), which attempts to balance the stoichiometry of the metabolites within the metabolic network with a goal (objective function) of maximal growth rate or maximal biomass accumulation. While flux predictions from FBA match experimental data reasonably well (Burgard and Maranas 2003), its assumptions may not always hold true, especially for GM or mutant lines. Several algorithms that have the goal of minimizing the change in the metabolic network upon perturbation have been developed, and appear to perform better than FBA in explaining fluxes of mutants (Segrè et al. 2002; Shlomi et al. 2005; Herrgård et al. 2006). This type of modeling could point to biochemical explanations for unintended or unexpected metabolite changes, which could help devise hypothesis-driven assessment strategies.

Using the genome-scale metabolic network of Arabidopsis, Rhee and colleagues tested the effect of single genetic perturbations of 136 genes (129 knock-out and 7 overexpression lines) by comprehensively profiling the metabolites using 11 analytic platforms including GC–MS, LC–MS, and ESI (Quanbeck et al. 2012). Comparison of the metabolite profiles across the mutants showed that metabolic networks were robust to perturbations of single metabolic genes and the genetic perturbations changed the network more locally than globally (Kim and Rhee, unpublished results). This study revealed relationships between characteristics of the perturbed genes and metabolic changes. More analyses of this type would help in identifying the relationships between changed metabolites and their potential impact on the metabolic system and biology of the organism, which in turn would inform if altered composition could have toxic or other harmful effects for food and feed safety in any given crop.

## Conclusions

The meeting summarized here was intended to present and discuss a broad range of viewpoints, rather than to arrive at a consensus on particular points. Nevertheless, several themes recurred in a number of presentations. For example, there seemed to be little disagreement with the observation that no system for genetic modification, including conventional methods of plant breeding, is without unintended effects. It was also commonly observed that “unintended” is not synonymous with “harmful”. The testing methods used to identify unintended effects from transgene introduction are based on analyzing the agronomic performance of the crop and composition of the harvested parts (e.g., grain, fruit, or forage). This testing, in combination with hypothesis-based testing of the effects and potential safety issues associated with transgene expression minimizes the likelihood of unsafe unintended effects associated with the GM crop products that are taken to commercialization.

The types of changes, such as genomic disruption caused by (trans)gene insertion, that were once seen as capable of leading to the production of unintended effects (e.g., in the form of novel toxins [Kessler et al. 1992]) turn out to be routine occurrences during all types of plant breeding and are ubiquitous in crop plants. Despite the ongoing presence of these changes during plant breeding and selection, there is not a single documented example whereby these changes have led to the production of previously unknown toxins. All reported cases of crop toxicity have been associated with the inadvertent elevation of known toxins, such that testing for their presence has become a part of the breeding process in some crops to prevent inadvertent increases in toxin levels (Steiner et al. 2013).

Emerging tools and resources such as genome-scale metabolic networks, quantitative network modeling, and metabolomics may help assess the effects of genetic modification on metabolism and may facilitate rational assessment of potential unintended effects of genetic modification on metabolism. The uses of networks and other omics-based technologies are still under assessment and are viewed as possible means of identifying potential unintended effects not tested by targeted approaches. As discussed during the meeting, there is not yet agreement as to whether the analytical and statistical methods currently available are sufficient to determine whether the profile of a new variety is meaningfully different (either globally or in the behavior of individual data points) from those of existing varieties.

## Electronic supplementary material

Below is the link to the electronic supplementary material.

Supplementary material 1 (PDF 294 kb)

## Footnotes

1 Section based on presentations from Wayne Parrott and Justin Vaughn.

2 Section based on presentation by Phil Bregitzer.

3 Section based on presentation by Mark Jordan.

4 Section based on presentations by Elena Rice, Esther Kok, and Phil Macdonald.

5 Section based on presentation by Laura Privalle.

6 Section based on presentation by Alan Raybould.

7 Section based on presentation by Alan Gray.

8 Section based on presentation by Andrew Bartholomaeus.

9 Section based on presentation by Jean-Michel Wal.

10 Section based on presentation by Thomas Holzhauser.

11 Section based on presentation by Elena Rice.

12 Section based on presentation by Paul Keese.

13 Section based on presentation by Jörg Romeis.

14 Section based on presentation by Esther Kok.

15 Section based on presentation by Seung Yon Rhee.
