# Supplementary material for: Genetic basis and detection of unintended effects in genetically modified crop plants
Source: Transgenic Res. 2015 Feb 26;24(4):587–603. doi: 10.1007/s11248-015-9867-7 (PMC4504983; doi:10.1007/s11248-015-9867-7)
Supplement: Supplementary file 1 — Supplementary material 1 (PDF 294 kb) [file 11248_2015_9867_MOESM1_ESM.pdf]

Submitted to Transgenic Research
Revised 22 February 2015

Online Supplementary Material

Genetic basis and detection of unintended effects in genetically modified crop plants

Gregory S. Ladics, DuPont Pioneer Agricultural Biotechnology, DuPont Experimental Station, 200 Powder Mill Road, Wilmington, DE 19803, USA

Andrew Bartholomaeus, Therapeutic Research Centre, School of Medicine, Queensland University, and School of Pharmacy, Faculty of Science and Education, University of Canberra, 52 Adamson Cres, Wanniassa 2903, Australia

Phil Bregitzer, US Department of Agriculture – Agricultural Research Service, National Small Grains Germplasm Research Facility, 1691 S. 2700 W., Aberdeen, ID 83210, USA

Nancy G. Doerrer, ILSI Health and Environmental Sciences Institute, 1556 15th St., NW, Washington, DC, USA

Alan Gray, Centre for Ecology and Hydrology, CEH Wallingford, Crowmarsh Gifford, Wallingford, Oxfordshire, OX10 8BB, UK

Thomas Holzhauser, Division of Allergology, Paul-Ehrlich-Institut, Paul-Ehrlich-Strasse 51-59, 63225 Langen, Germany

Mark Jordan, Agriculture and Agri-Food Canada, Cereal Research Centre, 101 Route 100, Morden, MB, R6M 1Y5, Canada

Paul Keese, Office of the Gene Technology Regulator, Australian Government, MDP54 GPO Box 9848, Canberra ACT 2601, Australia

Esther Kok, RIKILT Wageningen UR, P.O. Box 230, 6700 AE Wageningen, The Netherlands

Phil Macdonald, Canadian Food Inspection Agency, 1400 Merivale Rd, Ottawa, ON K1A 0Y9, Canada

Wayne Parrott, Center for Applied Genetic Technologies, University of Georgia, 111 Riverbend Road, Athens, GA 30602, USA

Laura Privalle, Bayer CropScience, 407 Davis Drive, Morrisville, NC 27560, USA

Alan Raybould, Syngenta Ltd, Jealott's Hill International Research Centre, Bracknell RG42 6EY, UK [†]

Seung Yon Rhee, Department of Plant Biology, Carnegie Institution for Science, 260 Panama St., Stanford, CA 94305, USA

Elena Rice, Monsanto Company, 700 Chesterfield Pkwy W., CC5A, Chesterfield, MO 63017, USA

Jörg Romeis, Agroscope, Institute for Sustainability Sciences ISS, Reckenholzstr. 191, Zurich 8046, Switzerland

Justin Vaughn, University of Georgia, Department of Genetics, 120 East Green Street, Athens, GA 30601, USA

Jean-Michel Wal, AgroParisTech, Dept. SVS, 16 rue Claude Bernard, Paris, 75231, France

Kevin Glenn, Monsanto Company, 800 N. Lindbergh Blvd, U4NA, St. Louis, MO 63167, USA

[†] Present address: Syngenta Crop Protection AG, Schwarzwaldallee 215, CH-4058 Basel, Switzerland

Corresponding Author:

Nancy G. Doerrer

ILSI Health and Environmental Sciences Institute

1156 15th St., NW

Suite 200

Washington, DC 20005, USA

ndoerrer@hesiglobal.org

Phone: 202-659-3306 x116

Fax: 202-659-3617

INTRODUCTION

In January 2014, an international meeting titled "Genetic Basis of Unintended Effects in Modified Plants" was held in Ottawa, Canada, bringing together over 75 scientists from academia, government, and the agro-biotech industry. The meeting was organized by the Canadian Food Inspection Agency (CFIA), the International Life Sciences Institute (ILSI) Health and Environmental Sciences Institute (HESI) Protein Allergenicity Technical Committee (PATC), the ILSI International Food Biotechnology Committee (IFBiC), the ILSI Research Foundation, and CropLife International. The objectives of the meeting were to explore current knowledge and areas requiring further study on unintended effects in plants and discuss how this information can inform and improve genetically modified (GM) crop risk assessments. The meeting featured presentations on (1) the molecular basis of plant genome variability, (2) unintended changes at the molecular and phenotypic levels, (3) a hypothesis-driven look at unintended effects in assessing conventional and GM crops, and (4) the consequences of unintended effects from a food and feed safety and environmental risk perspective. The extent to which unintended or unexpected changes pose a hazard was also discussed.

Following the summary of Phil Macdonald's introductory talk, this paper is divided into three broad areas: the molecular basis for unintended genomic changes in GM plants, observations and context for unintended changes seen in GM plants, and the interpretation of those changes for the purpose of safety assessment. Each section was provided by the indicated author shortly after the meeting, with new and updated information added by some authors since then. Presentations and other information from the meeting can be found at http://www.hesiglobal.org/i4a/pages/index.cfm?pageID=3654.

Unintended effects in genetically engineered plants: what they are and how they are assessed in Canada *(Phil Macdonald, CFIA)*

The Government of Canada recognized the need to develop a regulatory approach to biotechnology that was based on science and geared towards protecting the environment, human health, and safety, while allowing Canadians access the potential benefits. In Canada, the regulatory scope covers plants that possess characteristics or traits sufficiently different from the same or similar species, not merely those created using the tools of modern biotechnology. The

result is a trigger for regulatory oversight based on the presence of novel traits in the Canadian environment in general, and not just on plants modified through recombinant DNA techniques. As a consequence of the product based regulatory approach, in Canada the regulated plant is referred to as a plant with a novel trait (PNT), which can include products of genetic engineering as well as more conventional breeding techniques.

As an example of the application of the Canadian product based approach, a canola variety modified through the process of mutagenesis or conventional breeding with a related species not present in Canada or by the insertion of a new gene may lead to a canola line with a novel herbicide tolerance. In each instance, the resultant canola line triggers a safety assessment due to the presence of the novel herbicide tolerance trait where the environmental interactions of the new variety will be evaluated, including the possibility of gene transfer to the same or related species and any possible harmful outcomes evaluated.

In Canada, a Federal Framework for Biotechnology was established in 1993 to address new crop products as well as the impact of biotechnology on other commodities such as veterinary biologics, food, livestock feed, and pest control products. An important aspect of the Federal Framework was to establish the principle of using existing legislation and institutions instead of the development of a “Gene Act” or the establishment of a “Biotechnology Agency”. This approach is designed to take advantage of the knowledge and expertise that is already present in regulatory departments and agencies but applied to traditional products. It is also an acknowledgement that the Canadian Government policy considers that PNT crops should be considered as an extension of traditional breeding techniques.

Unconfined Release

The environmental risk assessment of PNTs is based on the concepts of familiarity and substantial equivalence. Familiarity applies to the species, the trait, and cultivation practices. Substantial equivalence refers to potential alteration of environmental interactions and considers the following five criteria:

- potential to become a weed of agriculture or be invasive of natural habitats

- potential gene flow to weedy relatives that may become weedy or invasive
- potential to become a plant pest
- potential impact on non-target species, including humans
- potential impact on biodiversity

The unconfined release assessment focuses on the potential interactions of the PNT with the managed agricultural ecosystem and the wider environment using the cultivated unmodified crop in Canada as the familiar comparator. The practice of comparing new or modified products with existing familiar products is a common approach for the safety assessment of plants derived through biotechnology. The Canadian approach is guided by the concept of substantial equivalence, as described by the Organisation for Economic Co-operation and Development (OECD 1993), to inform hazard identification and to give a scope for acceptable risk. The comparative assessment is used to determine whether there are significantly different or altered interactions with other life forms resulting from the PNT or its novel gene product(s) and whether the plant could become a weed of agriculture, become invasive of natural habitats, or be otherwise harmful to the environment. Recognizing that the production and management of a crop in agriculture has an effect on the environment including biodiversity, the PNT crop is considered in the same context. At the environmental level, the safety assessment will identify risks that could be posed by the new crop, including jeopardizing sustainable production, and determine if the risks posed by the modified crop are any greater than that posed by its unmodified counterpart. The novel trait, for example the production of a new protein produced by the plant, would be considered in the context of the gene products of the plant, recognizing that the proteins produced by the unmodified plant can have positive, neutral or negative environmental effects; does the new protein pose a greater risk? Experience in Canada has shown that while unintended effects can arise as an outcome of the genetic engineering, the phenotypic characterization is robust enough to capture any consequences for the genetic engineering and the risk assessment will identify whether the differences can result in a potential pathway to harm. Generally, unintended effects arising from the genetic engineering of a plant would be expected to include such phenomena as non-homologous end joining, recombination, deletions, and mutations, similar to the events that can also occur during conventional breeding.

Using familiarity and substantial equivalence as guide posts for the comparative safety assessment allowed the CFIA to authorize the first approval for a PNT in 1995. These approaches have proved robust enough allow Canada to continue to provide safe access to PNT crops, including those derived from genetic engineering. Canada's unique approach to the regulation of biotechnology that resulted in the PNT approach provides a broad perspective on all aspects of plant breeding, including using the modern tools of genetic engineering. Canada continues to evolve the regulatory oversight of PNTs, including how best to incorporate a consideration of the intended and unintended consequences of PNT production that respects our growing experience uses a weight of evidence approach and has an evenly balanced approach to the wide range of techniques that can be used to produce a PNT.

MOLECULAR BASIS FOR UNINTENDED GENOMIC CHANGES IN GM PLANTS

The molecular biology of gene function *(Mark Jordan, Agriculture and Agri-Food Canada)*
The effects of any given gene are determined by the structure of the gene and its flanking elements (see Watson et al. 2013). The coding sequence of the gene specifies the protein that is produced. Different proteins will have different effects due to their structure and function, with some proteins having many effects. A single gene that affects multiple traits is known as pleiotropy. The effects produced by a gene are in part regulated by other aspects of the gene structure such as promoter, 5′ and 3′ untranslated regions (UTR), Kozak sequence, and exon/intron boundaries. The promoter determines the timing and level of expression of the gene. This is accomplished via response element sequences that bind nuclear factors responsible for activating or repressing transcription. Similarly, factors present in the cytoplasm can bind to the 5′-UTR and stimulate or repress translation from the mRNA into protein. The protein produced from a particular gene is dependent on translation starting from the correct ATG start codon and stopping at the correct stop codon. The bases surrounding the ATG start codon, known as the Kozak sequence, are important in determining the ATG that is used as the start codon. The protein coding sequence itself is interrupted by non-coding DNA known as introns. The introns are removed from the pre-RNA to form the final messenger RNA (mRNA). There can be variation in the splice junctions used to create the mRNA that can lead to multiple versions of

mRNA, and therefore proteins, derived from a single gene in a process known as alternative splicing. The 3′-UTR is a major site of gene regulation as it can bind proteins governing mRNA stability and decay. A more stable mRNA can be translated into more copies of a protein than an mRNA that decays rapidly. The 3′-UTR is also the site of binding for microRNAs, which are small RNA molecules that when activated bind to the 3′-UTR and inhibit translation or target the mRNA for degradation, thereby reducing protein production (Li and Zhang 2013).

Mutations in these sequences can alter the way a gene is regulated resulting in changes in protein levels, structure of the protein itself, or changes in temporal or physical location of gene expression. This altered regulation is a result of changes to binding sites of regulatory proteins. In addition, different plant species or genotypes of the same species may differ in genes for regulatory proteins thereby modifying gene expression resulting in unanticipated effects. As it is possible to test early on for most of these changes to core gene regulatory regions, and given that some of these unanticipated effects are beneficial, such altered genes have been selected for by plant breeders.

Pleiotropy can be direct (where the secondary traits are derived from the primary trait) or indirect where one gene affects multiple traits independently. Indirect pleiotropy is more difficult to predict. Some of the traits affected can be beneficial from a plant breeding perspective and some can be negative. When both occur with a given gene, it is known as antagonistic pleiotropy. One such example is the wheat (*Triticum aestivum*) gene *Lr34*. This gene affects numerous traits providing durable resistance to a number of wheat diseases; however, it also causes premature senescence of the flag leaf (leaf tip necrosis), which can reduce potential yield in the absence of disease. If the wheat *Lr34* gene is moved into another species such as barley (*Hordeum vulgare*), the negative effect becomes stronger and the plants exhibit stunted growth and sterility (Risk et al. 2013). Clearly, wheat has regulatory mechanisms that control the expression of the gene in a manner that minimizes the negative effects of the gene. Variation exists in the amount of leaf tip necrosis among wheat genotypes but plant breeders have selected lines that maximize the benefit while reducing the negative effect of the gene. *Lr34* has been cloned (Krattinger et al. 2009) and the gene encodes an ABC transporter, which is a molecule involved in the transport of metabolites across membranes.

Prediction of whether pleiotropy (and therefore the possibility of unintended effects) is likely to occur depends on knowledge of the mechanism of action of the encoded protein. Genes affecting basic cellular functions that are needed by many traits (such as ABC transporters) increase the chances of pleiotropy being observed. Similarly, genes in which alternative splicing occurs in the pre-RNA or where the encoded product affects multiple pathways (for example transcription factors or other regulatory proteins or molecules) would also be more likely to produce pleiotropy.

The origin of the gene may also be an indicator of whether pleiotropy is likely to occur but this is harder to predict. There could be more pleiotropy if the gene originates from another species due to lack of regulatory controls (e.g., *Lr34* in barley) or less pleiotropy due to lack of a pathway/function in the new species compared with the original. In general, a gene that has multiple effects also has multiple controls, and the more closely related the recipient species is to the donor species, the more likely it is to have the controls. Variation in regulatory mechanisms is not only observed at the species level—different genotypes of the same species can have variation in regulatory mechanisms and result in different effects (what plant breeding is about). Prediction of unanticipated effects requires knowledge of gene function as well as of gene regulation. Knowledge arising from the complete genome sequence of crops will allow greater prediction of the effects of particular genes.

<u>The molecular biology of plant genomic changes and their role in unintended effects</u>

(Wayne Parrott, University of Georgia)

The advent of plant transformation technology brought with it concerns stemming from hazards presented by the transgene and its products, and protocols have been developed to evaluate these hazards.

Additional concerns come from the insertion of the transgene itself or from mutations that take place during the transformation process. As first articulated by Kessler et al. (1992), such mutations could deactivate repressors that are silencing metabolic pathways, leading to their

activation. The inadvertent activation of such pathways is a safety concern if the pathway leads to production of toxic metabolites not known to exist in the crop. The unknown nature and concentration of these novel metabolites makes it particularly difficult to determine their potential presence.

To illustrate the point, examples are known whereby insertions lead to production of metabolites in novel tissues, such as zeaxanthin in maize (*Zea mays*) kernels (reviewed in Weber et al. 2012) and anthocyanin in blood oranges (reviewed in Oliver et al. 2013). In these examples, a color change is associated with the change in metabolite production. However, if the metabolite was colorless, tasteless, and toxic, it could remain undetected until it caused harm to a consumer.
To better assess the possibility of inadvertent production of novel toxins, it is necessary to first evaluate the background levels of insertions and other mutations of concern in crop plants, and then to determine the extent to which these changes have resulted in the production of toxins that had not been known to exist in that crop.

The advent of high-throughput sequencing and other genomic technologies has made it possible to evaluate the nature and extent of naturally occurring genomic changes. These were extensively reviewed by Weber et al. (2012). Salient points reviewed include the following:

- Single nucleotide changes are common, with a background rate of seven new mutations per billion base pairs (bp) of DNA, or roughly, seven new mutations for every soybean (*Glycine max*) plant in every field.
- Insertions from transposons can be very common as well, with rates as high as 50 novel insertions per plant per generation reported in a variety of rice (*Oryza sativa*).
- Plants create novel genes through transposon capture, whereby pieces of different genes are assembled in novel combinations.
- There are genes that are present in different numbers or absent altogether in different individuals within a crop.
- Horizontal gene transfer is not uncommon, with pararetroviral sequences being particularly abundant in the genomes of crop plants.

The types of changes that were once seen as capable of leading to the production of unintended effects in the form of novel toxins (Kessler et al. 1992) turn out to be routine and ubiquitous in crop plants. Despite the ongoing presence of these changes during plant breeding and selection, there is not a single documented example whereby these changes have led to the production of previously unknown toxins. All reported cases of crop toxicity have been associated with the inadvertent elevation of known (as opposed to unknown) toxins, such that testing for their presence has become a routine part of the breeding process to prevent inadvertent increases in toxin levels (Steiner et al. 2013).

Also despite the prevalence of single-base-pair changes, no example is known where such a change has turned an innocuous protein into a toxin or allergen, as toxicity and allergenicity are properties that result from far more than the presence or absence of a given amino acid (Weber et al. 2012).

To summarize, the view of plant genomes as finely tuned gene networks that can be disrupted by changes in DNA simply cannot be supported with the available data. Instead, plant genomes are very dynamic and plastic, as predicted by Barbara McClintock (1984) in her Nobel address. Despite the ever-changing nature of plant genomes, the production of a previously unknown toxin in a crop has not been reported.

The biology of the plant genome makes it clear that there is no biological reason why the probability of obtaining harmful, unintended changes from plant transformation should be any different from the probability of getting the same harmful change during conventional plant breeding and selection. This latter probability has been too small to measure.

<u>What does it take to bring an agricultural biotechnology seed product to market?</u> *(Laura Privalle, Bayer CropScience)*

The extensive vetting process involved in the generation and selection of the “elite” event ensures that there are no safety issues associated with the GM crop products that are taken to commercialization. However, not all ideas lead to products. Sometimes, the trait is too complex;

the plant does not perform appropriately, i.e., yield or fertility could be impacted; or the economics are not favorable (that is, the value added cannot be captured or does not justify the expense of gaining global approvals). It is important to start big, working with many ideas, many traits, and many events, to allow identification of the single elite event that is worth seeking approval (Privalle et al. 2012). This is not unlike the approach taken by breeders searching for that new and improved elusive variety.

For GM crops, after efficacy (the most important factor), the main selection criteria applied in identifying the elite event are the molecular characteristics of the event, the thought being that the insertion site within the genome could have safety implications. The insertion should be present at a single locus and as a single copy. There should be no vector backbone present in the event and the insertion should not have disrupted an endogenous gene or created a chimeric novel fusion protein. There should be minimal locus rearrangement and the integrity of the gene cassette should have been preserved. None of these parameters have been demonstrated to impact the safety of the crop but are based on hypothetical, minimal-probability possibilities. Since most GM products require global approvals, the strictest restrictions, regardless of geography, dominate the event selection criteria.

Once the elite event is identified, an extensive safety assessment is conducted that includes studies on the safety of the newly expressed protein, molecular characterization of the insert, impact of the insert on plant performance and composition, environmental impact, and wholesomeness of the crop (Codex 2003). GM foods are the most highly studied foods consumed. The registration dossiers are scrutinized by regulatory agencies around the world. The approval for any event has not been withheld based on any unintended effect of the insertion of the gene cassette within the genome.

However, not all products are successful and not all approved events are actually commercialized. Examples of product ideas that were considered but never commercialized include high-methionine soybean, produced by introduction of the 2S protein from Brazil nut (Nordlee et al. 1996); this protein was identified as the Brazil nut allergen and is frequently used as an example of how well the system works at deterring unsafe products. Another example of

this would be the bean amylase inhibitor protein in pea (Prescott et al. 2005). There are many examples for vegetables, including nutritionally enhanced tomatoes with higher lycopene or other anti-oxidants; and β-carotene in sweet potatoes. The market cannot support the regulatory costs of these products.

The first GM product to receive approval, FlavR SavR tomato, was never financially successful and although customers wanted it, it was not commercialized for very long (Martineau 2001). Maximizer™ Knockout Bt Corn was the first maize product to receive regulatory approvals (even for cultivation in Europe) but was replaced on the market by other products (Altman and Hasegawa 2011). Triffid flax, containing an herbicide tolerance trait, produced by the University of Saskatchewan, was the first example of a federally approved crop being removed from the market without being obsolete or defective (Ryan and Smyth 2012). Bt potato (New Leaf Plus ®), which provided an effective, green solution to the Colorado potato beetle pest (Perlak et al. 1993), was withdrawn in 2001 because of public perception issues. Amflora potato (high-amylopectin starch), after 14 years of evaluation by regulatory authorities, received approval in March of 2010. It was only marketed for a single season despite the huge benefits to both the environment and the potato starch industry in reducing energy required for amylopectin starch production.

In summary, it takes many tries to produce the elite event for commercialization. Plant performance and efficacy together with characterization of the insertion are used to identify the elite event. The safety assessment presented in the dossier to regulatory agencies includes the molecular characterization and confirmation of plant performance among many other studies. These studies are reviewed by regulatory agencies around the world. The failures of products have not been a result of safety issues but rather the result of economic or public perception issues. No product has been withdrawn or not approved due to an unintended effect related to insertion into the genome.

Avoid and remove unintended changes with "Clean Gene" delivery and breeding strategies

(Phil Bregitzer, USDA-ARS)

A good GM plant, from the perspective of a plant breeder, is one in which a limited, predictable change has been made that improves plant performance. For instance, a commercially useful barley cultivar for malting and brewing will combine high yield potential with a specific set of biochemical characteristics. This performance results from selective breeding that has assembled particular allelic combinations that interact to produce the desired performance. Ideally, genetic modifications via non-sexual introduction of DNA should not disturb this carefully assembled genome, but should result only in the limited and predicted changes caused by the introduced DNA.

Creating transgenic plants depends on mutagenic in vitro environments, suffers from imprecise transgene insertions, and employs selectable markers. Markers are used intentionally, while the creation of aberrant transgene loci and somaclonal variation (SCV; tissue-culture-induced mutation) are unintentional, but all are related by the common thread of undesirability in the final product.

Since the culture of semi-differentiated plant tissues in vitro is critical for most transformation schemes, an obvious impediment to preserving the genetic integrity of the plant to be transformed is SCV, or tissue-culture-induced variability (Larkin and Scowcroft 1981). SCV is a ubiquitous phenomenon in all plant tissues cultured *in vitro*. Although SCV is potentially useful as a source of novel mutations, it is antagonistic to the objective of making limited, predictable changes as a result of transgene introduction. Significant and negative changes have been noted in the agronomic performance and malting quality of tissue-culture-derived plants. Yield losses of 15–84% have been observed in non-transgenic derivatives of transgenic plants (Bregitzer et al. 1998). Although certain adjustments to the *in vitro* environment can reduced the severity of somaclonal variation, the most effective way to eliminate it in barley has been to backcross transgenic plants to plants without any SCV. Backcrossing is a process of making repeated crosses to a recurrent parent (such as the wild type parent used in making the original transgenic plant) with selection at each generation based only on the presence of the transgene. Backcrossing will ultimately replace most of the genome of the transgenic plant with that of the recurrent parent, thus eliminating variant alleles that were present in the transgenic plant, with the exception of alleles at closely-linked loci. In practice, a single backcross to Conlon recovered

the majority of yield loss caused by SCV in a group of transgenic lines that were created using Conlon. On average, the yield loss in the primary transgenic lines was 31%, versus 6% in the backcross-derived lines (Bregitzer and Dahleen 2008).

Transgenic loci can suffer from rearrangements—including inverted repeats that can induce transgene silencing—and are usually accompanied by selectable markers used for selection in vitro, and by bacterial DNA derived from the cloning vector. Transgene delivery methods more likely to deliver intact transgenes (e.g., *Agrobacterium* vs. biolistics) are advantageous, but don't address the problem of linked selectable markers and bacterial vector backbone sequences. An ideal delivery system would deliver "clean" transgenes that are intact, single copies without linked selectable markers or vector DNA. One option that will enable this involves harnessing the power of DNA transposable elements, or transposons, which are able to move from one place in the genome to another—the process of transposition—as an intact unit. The *Ac*/*Ds* (*Activator*/*Dissociation*) system derived from maize, first described in the 1940s, has been intensively studied and is relatively well-understood (Fedoroff 1989). The *Ac* transposon has two critical features: the *Ac* transposase gene, and terminal sequences that interact with the transposase protein to induce transposition. *Ds* transposons do not have a functional *Ac* transposase, and can range from nearly-intact *Ac* transposons to highly-modified derivatives, with the only restriction being the retention of short termini recognizable by *Ac* transposase. This feature enables the construction of synthetic *Ds* transposons by flanking a DNA fragment, such as a transgene expression cassette, with these termini. For barley, synthetic transposons have been built that utilize terminal sequences of 251 and 317 bp—relatively short sequences that do not create significant problems for vector development or introduction into plants.

Using transposition to deliver transgenes with our system is a two-step process. First, a transgenic cassette with a *Ds*-bordered gene of interest and a selectable marker, inserted into a cloning vector, is introduced into the plant. This construct differs from standard constructs only by the presence of the *Ds* termini around the gene of interest, and the initial transgene locus in the resulting plant will be typical—that is, it will include the selectable marker, vector DNA, and be subject to rearrangement. The second step is hybridization to a plant expressing *Ac* transposase to induce transposition, which will move the *Ds*-bordered gene to another, often

unlinked, location. Genetic segregation and selection among progeny will enable isolation of plants without the original transgenic locus, but with a transposed, single-copy intact transgene that is—in the majority of cases—located in regions that support good transgene expression (Bregitzer and Brown 2013).

Delivery by transposition contributes to predictability by delivering intact transgenes with known borders. This facilitates sequence-based transgene mapping, which enables selection against plants in which transgenes inadvertently are located in or near native genes. Furthermore, transgene expression is not compromised by rearrangements, and the potential for pleiotropic effects caused by selectable marker expression is eliminated.

No system for genetic modification, including the time-tested, generally regarded as safe, traditional methods of plant breeding is without unintended effects. Thus, it is imperative that new crop varieties are carefully evaluated for negative characteristics. Modern plant breeding has incorporated robust testing methods that ferret out problems with the background genome by analyzing agronomic performance and qualities of the harvested parts (grain, fruit, or forage). In combination with hypothesis-based testing of the effects and potential safety issues associated with transgene expression, transposon-based gene delivery combined with proper breeding procedures such as backcrossing, followed by appropriate testing, can produce plants with minimal unintended effects.

<u>How much of a hazardous substance would have to be produced before it poses a risk?</u>

(Andrew Bartholomaeus, Queensland University, University of Canberra)

To address the question in terms of unintended effects in biotechnology developed crops, the context within which it is to be answered must be defined. From a regulatory perspective, the appropriate context is the range of plausible scientific possibilities, after excluding the many highly speculative theoretical excursions that lack concrete examples, despite the rapidly expanding data that is available on natural compositional variability and the plasticity of the plant genome. To identify the boundaries defining the context therefore one must first EXCLUDE hypothetical, extreme or scientifically implausible circumstances, IDENTIFY

practical scientifically plausible possibilities and probabilities based on the extensive data now available, and DEFINE the nature of the at risk group(s) to be addressed (population or individual health risks), the type of hazard(s) of concern (toxicological, dietary, immunological), and the risk time metric (acute, sub-acute, or chronic).

The de novo production of systemically toxic proteins unrelated to the parent plant variety or the function of the transgene can be excluded as implausible, because systemic toxicity of an ingested protein requires at least three highly specific, and separate, structural characteristics. These are resistance to digestion, ligand specificity for gut uptake transporters, and ligand/receptor specificity for site- and species-specific receptor-mediated toxicity. These requirements are implausible outcomes from plant breeding individually, and essentially impossible collectively. Similarly, the potential for random genome effects to modify existing non-toxic proteins to create a toxic protein is also essentially zero as evidenced by the current knowledge of non-GM maize and other food crop varieties that have millions of single-nucleotide polymorphisms (SNPs) across varieties but have never resulted in a toxic protein in a food crop. The de novo generation of the machinery necessary to produce a toxic secondary metabolite can be excluded because of the multiple coordinated changes in plant biochemistry that would be needed and the absence of such an event in the extensive range of varieties produced by genetic manipulation in conventional and GM crop breeding over the past century. The reactivation of dormant pathways has also never been observed and is implausible due to the accumulation of mutations in non-functional DNA, progressively degrading any residual potential functionality.

In common with conventional plant breeding, plausible possibilities for compositional alteration of toxicological relevance include the up (and down) regulation of pre-existing endogenous plant toxins, increased/decreased uptake of minerals from the soil or water (e.g., Cd, As, Se), altered levels of in nutrients or antinutrients associated with population health outcomes, altered production of pesticide metabolites, altered levels of toxic substrates (precursors) due to blocking of an enzyme pathway, and altered release/availability of endogenous toxins.

In considering the extent of compositional alteration necessary to be of regulatory interest, the primary comparator is the natural variability of the substance both in the parent crop variety that has been modified and in the range of crops that contain that substance.

The threshold levels for concern will depend on the nature of the risk exposed group of interest. If the focus is population health, smaller levels of risk may be of concern than for an individual, and legitimate population risk may not be relevant for specific individuals without an individual clinical assessment. Individual safety issues are generally concerned with more immediate, direct, and substantial risks likely to affect most individuals exposed. The type of hazard involved is another important consideration in terms of risk management and also in considering investigational approaches. Toxicological risks are primarily a function of the nature of the chemical of interest with individual variability only a relatively minor factor. There is generally a good level of cross-species similarity of effect and consequently animal studies are useful and generally predictive, although with caveats. Immunological or idiosyncratic risks such as allergy or intolerance are primarily a function of the individual, with chemical characteristics necessary but not sufficient in isolation. There is at best poor cross-species correlation and consequently animal studies are generally of marginal value. Dietary risks are primarily a function of individual dietary patterns and again the chemical characteristics although necessary are not sufficient and animal studies generally of very limited value (Hayder et al. 2011).

From the discussion above, endogenous secondary metabolites are a clear potential candidate for alteration in levels and a number of examples exist of their up or down regulation. These compounds are produced by plants as a means of responding to specific pest pressures (Bourgard et al. 2001). Their toxicity is not therefore random. Digitalis glycoalkaloids from foxglove, for example, are specific cardiac toxins in mammals and are a cell-membrane-damaging surfactant for microorganisms, thus providing protection against herbivores and microbial pests (Wink 2010). The levels of secondary metabolites are known to be highly variable, in response to a range of stressors such as pest pressure, agronomic practices, climatic variables, and pollution. Plants produce secondary metabolites as natural pesticides and consequently they are ubiquitous in the human diet, many being intentionally added to food as culinary flavorings. In considering the risks presented by these compounds highly sensitive, but poorly specific, study designs such

as the lifetime rat study need to be considered in a broader risk context. Of over 70 secondary metabolites studied in rats, for example, over half were carcinogens in the rat. These results have poor relevance to humans because of the extreme study conditions (Ames and Gold 2000). Other secondary metabolites such as aristolochic acid, found in many Chinese traditional medicines, are frank, potent, human carcinogens after even short term exposure to relatively modest doses however. Aristolochic acid in traditional medicines has been linked to upper urothelial carcinoma in a number of countries including Taiwan, which has the world highest incidence of this carcinoma (Chen et al. 2012).

Plausible compositional alterations of toxicological interest in crops developed through both conventional and biotechnology techniques will relate to the physiology of the parent plant variety and the inserted transgene. Although some of these changes may be unintended, they are generally not unexpected and the principle means of identifying them is targeted compositional analysis with comparison against the natural range of variation in the substance of interest under diverse agronomic, climatic, environmental, and pest influences. How much constitutes a concern depends on the context of the question and the normal background variation as much as the nature of the substance itself.

OBSERVATIONS AND CONTEXT FOR UNINTENDED CHANGES SEEN IN GM PLANTS

<u>The biology of naturally occurring insertions</u> *(Justin Vaughn, University of Georgia)*
Genetic engineering in plants is typically mediated by the insertion of a modified T-DNA sequence from *Agrobacterium tumefaciens* into the genome of a crop. This insertion can disrupt the function of native genes and can create rearrangements at the site of insertion. The genomic coordinates of a T-DNA insertion and its position relative to native genes can be determined exactly. In addition, the sequences flanking the T-DNA insertion can be sequenced to ascertain if any unanticipated structural changes have occurred as a result of integration. Roughly half of insertions exhibit less than 8 bp of “filler” DNA at the junction site, while the other half of insertions contain larger additions, generally between 8 and 100 bp (Forsbach et al. 2003).

Whether such "filler" sequences contain functional elements and the degree to which those elements are active would require case-by-case analysis. The objective of this summary is to contrast such events with the frequency and severity of structural changes that we would expect in non-regulated, conventionally bred varieties.

New sequencing technologies are allowing scientist to interrogate the degree of structural variation in a population at a scale unprecedented a decade ago. By re-sequencing numerous Arabidopsis genomes, researchers showed that after a single generation roughly 3 in every 100 plants of the exact same starting genotype will have a deletion involving over 50 bp of DNA (Ossowski et al. 2010). Given the technical limitations at the time, both insertions and transposable element activity were likely undetectable in that study; therefore, these data represent a lower limit to the rate of such mutations. Indeed, an earlier study using longer Sanger sequencing reads contrasted two rice varieties and showed that insertions resulting from double-strand break repair and transposable element movement account for much more DNA rearrangement than deletions (Ma and Bennetzen 2004).

In order to understand their origin and frequency, we have recently explored these structural variations at the population level in rice (Vaughn and Bennetzen 2014). Such variation is clearly common, even in closely related varieties. We can compare these data with results from experiments that directly assay the structural repercussions of double-strand break repair (Kwon et al., unpublished). This comparison indicates that such repair events are the source of the structural variations described above and that the "filler" DNA observed in T-DNA insertion has a clear precedent with regard to naturally occurring mutations.

In conclusion, genetic transformation has the potential to disrupt or rearrange pre-existing genes. Though such events can change crop quality and composition, they commonly occur in natural populations and across non-transgenic cultivated varieties. Indeed, the scale of rearrangement in a transgenic plant is likely to be far less extensive than commonly observed between two distinct varieties. In addition, the types of rearrangements that we see at T-DNA insertion sites are similar to those resulting from the repair of double-strand breaks that continuously occur in natural and breeding populations. These conclusions are just as applicable to emerging

techniques that exploit site-specific double-strand break repair for engineering purposes (Chen and Lin 2013).

<u>Unintended effects on allergens in carrots and apples</u> *(Thomas Holzhauser, PEI)*

Allergies to fruits and vegetables affect approximately up to 4 % of the population in Europe (Zuidmeer et al. 2008). Carrot (*Daucus carota*), a vegetable of the Apiaceae family, and apple (*Malus domestica*), a fruit of the Rosaceae family, are among the most prevalent elicitors of allergic reactions to foods in northern and central Europe. Allergy to apple and carrot is frequently observed in relation to birch pollen allergy. Additional co-reactivity to mugwort pollen is found in carrot allergy.

Both allergenic foods contain pan-allergenic structures, i.e., proteins that are widely distributed throughout the plant kingdom and which may be responsible for allergic cross-sensitization and reactivity, even between phylogenetically unrelated plant species. Examples are apple Mal d 1 and carrot Dau c 1, which are both homologous allergens to the major birch pollen allergen Bet v 1. In addition, profilin has been described in apple (Mal d 4) and carrot (Dau c 4). Further, non-specific lipid transfer protein (Mal d 3) and a thaumatin-like protein (Mal d 2) have been described in apple, and isoflavone reductase and cyclophilin in carrot. Several of these pan-allergens belong to pathogenesis-related protein families (PR-5, PR-10, and PR-14), which are considered stress-inducible, or to other protein families that also may be involved in plant defense mechanisms. Thus, genetic factors, such as cultivar diversity, and environmental factors, such as the conditions of cultivation, harvest, and post-harvest storage, may have an impact on the qualitative and quantitative composition of allergens and isoallergens in apples and carrots.

Published studies on the allergenicity of apple and carrot so far have focused on non-transgenic cultivars. The study objectives included the investigation of changes in allergen and isoallergen profiles, the identification of low allergenic cultivars, and the association of allergen composition to in vitro allergenicity and in vivo clinical reactivity in allergic subjects. In addition to the initial objectives, the available data on isoallergen expression and composition of the non-transgenic apple and carrot cultivars help increasing our knowledge about the natural variation in allergen

and isoallergen composition as well as potential pathways of allergen and isoallergen expression. Further, this knowledge may form a basis towards hypothesis-driven studies, e.g., to investigate intentionally modified biochemical pathways of allergen expression in transgenic lines, as well as to define the range of natural variation as a benchmark for the transgenic counterpart.

Most of the available data on apple allergen quantification and allergenicity relate to the previous European funded project 'SAFE' (QLK1-CT-2000-01394), where apple was chosen as a model because of the high prevalence of apple allergy in Europe, its importance as a crop, and the observation that the clinical reactivity tends to vary between different geographic regions in Europe. The latter observation has been linked to differences in sensitization profiles, such as a higher prevalence of sensitization to Mal d 3, accompanied by more severe allergic reactions to apple in the Spanish compared with the central or northern European study population. Furthermore, the allergenicity of twenty-one apple cultivars was assessed by skin-prick testing (SPT) in fifteen Dutch patients with a birch pollen-related and Mal d 1-mediated apple allergy (Bolhaar et al. 2005). Apple cultivars were grouped according to the SPT wheal size, and significant differences were found between apples of low versus high SPT reactivity. In a subsequent double-blind placebo-controlled food challenge in five Dutch patients, 'Golden Delicious' and 'Santana' were further chosen as examples of cultivars with high versus low SPT reactivity. With statistical significance, the cultivar 'Golden Delicious' presented an approximately thirty times higher allergenicity with regard to symptom severity than 'Santana' (Bolhaar et al. 2005). A molecular analysis of the gene expression of seven intron-containing genes in fourteen apple cultivars further suggested a positive association of the genes of Mal d 1.04 and Mal d 1.06A with SPT reactivity (Gao et al. 2008). In another recent study, the expression profiles of the so far known Mal d 1 genes were investigated in the cultivars 'Florina' and 'Gala'. Of the thirty-one investigated Mal d 1 genes, twenty were expressed in the fruit. Partially large differences in transcription rates were found between the studied cultivars, the tissue (peel versus flesh), and the various transcribed genes of the isoallergens (Pagliarani et al. 2013). On the protein level, the influence of a five-month post-harvest storage on the quantitative level of Mal d 1 and Mal d 3 allergens was investigated in another two studies. With allergen specific antibody-based quantification, an increase of up to twenty-fold was found for Mal d 1,

and a decrease down to one tenth of the initial amount was found in Mal d 3 in the selected apple cultivars (Sancho et al. 2006a, 2006b).

In a study that focused on the allergen composition of carrots, the isoallergens Dau c 1.01 and Dau c 1.02 were quantified using ELISA (Foetisch et al. 2011). Two cultivars, 'Rodelica' and 'Nerac', were investigated in a two-year follow-up study. A first evaluation of the field data suggested a large influence of the year of cultivation and an apparent difference between the two cultivars (unpublished data, research project BÖL 03OE349 granted by the German Federal Ministry of Food, Agriculture and Consumer Protection).

In summary, the presented data suggested that apple and carrot can be considered model foods to study the influence of genetic and environmental factors on the composition of pan-allergenic structures and the isoallergen distribution in fruits and vegetables. With regard to clinical reactivity, some isoallergens might be of greater relevance than others. The level of allergens can increase or decrease depending on genetic and environmental factors. Finally, the knowledge about biochemical pathways of allergen translation and about the range of natural variability of the allergen composition may form a basis towards hypothesis-driven studies on unintended effects in modified plants intended for human consumption.

Single-gene traits *(Alan Raybould, Syngenta)*

Risk is a combination of the seriousness and likelihood of a harmful effect following a course of action. Risk assessment characterizes the amount of risk associated with an activity. It contributes to making decisions about whether to undertake an activity, such as the import, field testing, or cultivation of specific GM crops. There is concern (e.g., Craig et al. 2008) that the amount of data required for risk assessments of GM crops is increasing and becoming detrimental to decision-making in many countries.

A significant contributor to increasing data requirements is a mistaken idea of how risk assessments ought to be conducted. In the “bucket” approach to risk assessment (Raybould 2011), data on the properties of the GM crop are collected in an untargeted (“unbiased”) manner,

often termed profiling. Profiling could comprise measurements of the crop's gross phenotype, its composition, its mRNA, its proteins, its metabolites, and so on. By comparing these profiles with those of a suitable non-GM crop, the risk assessor is supposed to be able to identify changes in the GM crop that are potentially harmful.

This approach to risk assessment is ineffective and inefficient. First, what to regard as harmful is defined by policy; it is not discovered in data (Sarevitz 2004; Sanvido et al. 2012). Secondly, even if harm is defined, profiling will collect many data that do not predict the seriousness or probability of harm following use of the GM crop. These data are irrelevant for risk assessment and may impair decision-making because they distract from data that are relevant.

A different approach regards risk assessment as hypothesis testing. The risk assessor identifies the harmful effects from relevant legislation or regulations (Evans et al. 2006) and builds scenarios comprising a series of events leading from the proposed use of the particular GM crop to the identified harmful effects. These scenarios, or "pathways to harm", allow the risk assessor to devise testable hypotheses about the likelihood, frequency or magnitude of the events in the pathway. Data are collected to test these hypotheses and thereby characterize risk (Raybould 2011). Targeting data collection on hypothesis testing is far more effective and efficient than collecting bucketsful of data of unknown relevance.

An imaginary example illustrates the difference between profiling and hypothesis testing. Say there is concern that cultivating GM maize producing an insecticidal protein may harm an endangered butterfly: the protein targets Lepidoptera and may be toxic to the species of concern. The habitat of the butterfly is alpine meadows, many miles from any maize cultivation. In order for the GM maize to harm the butterfly, several events are necessary: GM maize seed must be dispersed into the butterfly's habitat; the seed must survive the winter; it must germinate and establish in the alpine meadows; butterflies must consume enough GM maize to suffer adverse effects; and the adverse effects must be sufficient to reduce the abundance of the butterfly.

It may be well known that non-GM maize seed cannot survive the winter frosts in the alpine meadows. A simple way to characterize risk to the butterfly is, therefore, to test the hypothesis

that the GM maize seed has not acquired greater frost tolerance than non-GM maize seed through some unintended effect of transformation; this could be done easily by freezing the seed for a few days and then attempting to germinate the seed at room temperature. If the hypothesis is corroborated, one could conclude that there is minimal risk to the butterfly from cultivating the maize. Indeed, one may regard even this test as superfluous: the whole scenario is implausible if maize seed does not disperse to the meadows.

It might be argued that if untargeted phenotypic or molecular profiling of the GM and non-GM maize found few differences one would be reassured that the risks posed by the GM crop are no greater than those posed by the non-GM crop. In other words, if the profiling data show no unintended effects of transformation then they corroborate the hypothesis that there are no potentially harmful unintended effects of transformation. Furthermore, one would have assessed risk objectively, and not have been "biased" by "assumptions" about how harm may occur. There are serious problems with the above argument. Profiling is not exhaustive: there are infinite endpoints and conditions that are not measured. Hence, finding no difference in the profiles does not mean that the hypothesis of no potentially harmful unintended effects is corroborated. The conditions of the experiment and the endpoints measured may be unable to detect potentially harmful unintended effects: the hypothesis is untested because it could not be falsified, especially if harm is undefined.

If differences are found, after many years' research we may find that a difference in concentration of a metabolite is a predictor of increased cold tolerance of seeds. After more research we may find that this increased tolerance allows maize seeds to survive in alpine meadows. Then we may wonder about the ecological effects of this increased survival and alight on possible adverse effects on a rare butterfly. Thus to give meaning to an "unbiased" method, one must postulate the pathway to harm that a targeted, hypothesis-driven ("biased") approach would have done, only spending significantly more time and effort producing many irrelevant data getting there.

Unlike like basic research, risk assessment cannot be unbiased. Effective risk assessment involves choosing which of the infinite possible effects of using a GM crop to be concerned

about. Our limited resources are then targeted on testing hypotheses about the probability and consequences of those effects. These will be strong tests of clear hypotheses, which, if corroborated, provide high confidence in conclusions of low risk. Unbiased profiling at best provides weak tests of vague hypotheses, giving little confidence in our conclusions about risk.

Hypothesis-driven evaluation of drought-tolerant maize in safety assessment *(Elena Rice, Monsanto)*

Plant breeders have successfully improved maize grain yields despite sometimes having very limited information on the genes and gene networks that impact grain yield. Genetic gains in grain yield have been possible largely through 1) creating novel gene combinations that affected the complex gene networks and 2) a careful selection process for yield. It is now clear that improvements in grain yield have been associated with significant changes in many other traits (Tollenaar and Lee 2010) and the exact function of the combined genes are mainly not known. Biotechnology can aid breeding to continue to increase grain yield through the introduction of specific genes with expected effects on endogenous pathways and phenotypes. However, the use of biotechnology-derived traits to advance yield gains has been viewed by some skeptical observers as a potential unique source of "unintended effects". The fact that this has proven not to be the case, is driven by several basic considerations:

- Breeding and biotechnology products undergo a similar process of selection for intended characteristics and elimination of undesirable phenotypes.
- Commercially viable biotech traits are selected to introduce small incremental changes in well-established phenotypes known to be associated historically with yield gains in conventional crops.
- Commercial biotech-produced candidates undergo a detailed safety assessment that includes molecular characterization, physiological assessments, metabolite and compositional studies, and extensive field testing.

An understanding of the mode of action of the inserted gene can help to evaluate the potential unintended effects that may arise from gene insertion and limit the data requirements to a

hypothesis-driven evaluation of the effects on plant phenotypes, physiology, and metabolites instead of exhaustively assessing all possible measurable phenotypes.

Recently Monsanto introduced DroughtGard® hybrids, which combine germplasm selected for superior drought tolerance and high yield potential with the world's first drought-tolerance biotechnology trait for maize. Biotechnology-derived event MON 87460 expresses bacterial cold shock domain protein B (*Bacillus subtilis* CSPB), which imparts reduced yield loss under water-limited conditions compared with conventional maize (Castiglioni et al. 2008). The CSPB in MON 87460 belongs to the cold shock domain–containing (CSD-containing) protein family, which has been extensively studied in bacteria. Under environmental stress, CSD-containing proteins have been shown to moderate stress responses in bacteria and plants primarily through stabilization of RNA and improved cellular function (Cristofari and Darlix 2002; Chaikam and Karlson 2008). Like endogenous CSD proteins found in bacteria and plants, we demonstrated that the CSPB protein in MON 87460 interacts with RNA and accumulates and localizes to rapidly growing tissues and in developing reproductive organs, thereby helping to maintain normal cellular function in those tissues (Nemali et al. 2014). Under water-limited conditions, there is a trend toward improved ear growth rate for MON 87460 compared with the control plants, while the common mechanisms of plant response to drought stress are not altered in transgenic CSPB-expressing maize plants (Castiglioni et al. 2008; Nemali et al. 2014). When plants were grown under well-watered conditions, no appreciable difference in these mechanisms between CSPB- expressing lines and the control were detected (Castiglioni et al. 2008).

Based on the understanding of the CSPB mode of action, the environmental risk assessment for MON87460 included six hypothesis-driven studies that answered specific questions relevant to the nature of the trait in addition to the standard phenotypic and agronomic field trials in the presence and absence of the trait (Sammons et al. 2014). The studies included assessments for persistence outside of cultivation; root growth and development; and drought, cold, heat, and salt tolerance (Sammons et al. 2014). No additional abiotic stress tolerances were identified and no differences in season-long water consumption or root growth and development were observed. These studies confirmed a lack of potential for any adverse environmental impact.

Thus, expression of the inserted CSPB protein in maize showed no evidence for pleiotropic effects associated with the improved plant response to water-limited conditions, no altered growth habits under a variety of well-watered conditions, and no increased pest potential when compared with a conventional control. An understanding of the mode of action could provide a context to develop risk assessment and hypothesis-driven evaluation studies addressing product safety.

INTERPRETATION OF UNINTENDED CHANGES FOR THE PURPOSE OF SAFETY ASSESSMENT

Identification / assessment of possible unintended effect(s) on the overall allergenicity of GM plants *(Jean-Michel Wal, AgroParisTech)*

The strategy for the safety assessment of GM plants is based on the comprehensive agronomic/phenotypic and compositional comparative analysis of the GM plant and its conventional counterpart in order to evidence possible significant and meaningful differences due to unintended effects of the genetic modification. In line with this strategy, when the recipient of the genetic modification is a known allergen (e.g., soybean), a comparison of the qualitative and quantitative composition in endogenous allergens should be performed (Metcalfe et al. 1996).

The concentration of endogenous allergens is highly variable and the comparative analysis should take into account the influence of the cultivars and of the conditions of cultivation, harvest, storage and processing on the expression of allergens. To allow relevant comparison the European Food Safety Authority (EFSA) guidance and the European Commission (EC) regulation have recommended including “key” endogenous allergens in the list of compounds to be determined within the compositional analysis performed on the plant materials collected from field trials. This aims to provide indications to assess whether the GM plant is more allergenic than its conventional counterpart (EFSA 2011; EC 2013). “Key allergens” are well-identified allergens that are relevant for public health because of their allergenic potency and abundance. They are generally well conserved proteins with important metabolic/physiologic functions for

the plant, such as enzymes, defense proteins, or storage proteins. Any significant difference could thus be directly related to the specific allergy risk but also indicate possible occurrence of other unintended effects of different nature.

Where necessary, the qualitative and quantitative analysis of “key allergens” is performed using profiling technologies. Most commonly used are targeted analyses using immuno assays. They generally consist of Enzyme Allergo Sorbent Tests on whole-plant protein extracts or western blotting in combination with 1- or 2-dimension gel electrophoresis (2-DGE) for separation of proteins according to their molecular mass and isoelectric point ; the detection of allergens is then made by incubation with specific sera from relevant allergic humans. They may present a high individual variability and heterogeneity in their specific IgE response that results in allergen patterns that greatly differ by their complexity. Each allergen often appears as a group of several spots of different isoforms and variants and confirmation of the identification using mass-spectrometric (MS) analysis is normally required for a complete interpretation. Those studies are relevant and informative but the selectivity of the method is an important point to consider to avoid pitfalls. Also, individual sera should be used instead of pooled sera in order to avoid the risk of overlooking minor allergens because of dilution of specific IgEs present in rare patients only and the availability of sufficient relevant sera may be a drawback.

Non-targeted analyses such as proteomic approaches using mass spectrometry, e.g., MALDI or ESI-TOF MS in combination with different separation methods such as 2-DGE or liquid chromatography, have been rapidly developed in the recent past years. They may not require human sera and have proven to be efficient (alternative) tools for the identification and quantification of known allergens in plants although it is still sometimes considered they may be complex and insufficiently standardized and would need further developments and validation before they can be routinely used for safety assessment.

The assessment of the biological relevance of a significant difference (e.g., overexpression of [some] endogenous allergens) that would be observed in a GM plant is based on several criteria such as the number and identification of concerned allergens and their importance, the magnitude of the differences, and whether those differences are incidental or observed in every season or

location of the field trials. The question on whether there is a (causal) relationship between increased expression of endogenous allergens, increased allergenicity of the GM food, and increased risk for allergic consumers is still a matter of debate, mixing arguments pertaining to risk assessment and risk management (Fernandez et al. 2013; Goodman et al. 2013). A multi-center study on the prevalence of sensitization to oilseed rape and maize pollens carried out in France by the Allergo-Vigilance Network provided an interesting insight on the relationship between exposure and risk of sensitization. It showed that

i) frequent sensitizations to pollen allergens were observed in atopic patients (and atopic patients only) living in regions that contain numerous rapeseed and maize fields,
ii) the incidence of sensitization was positively correlated to the level of exposure (i.e., to the crop density),
iii) the prevalence was higher in patients with actual atopic disease as compared with those with asymptomatic atopy, and
iv) cross-reactivity observed between pollens and seeds could potentially elicit cross-reacting food allergies (Moneret-Vautrin et al. 2012).

Therefore, significant differences in the pattern of expression of endogenous allergens may require additional information/investigations to further characterize the allergenicity of the GM plant.

It should be noted that to date, those considerations concern only GM soybean and that no significant differences between GM soybean and conventional comparator have been observed in applications assessed so far. Generally differences are much bigger between reference lines than between GM and non-GM comparator. Of course, this is not an absolute rule and it should be checked and confirmed in each case. However, depending upon evolving epidemiological data or clinical case reports collected in new geographic areas, this may apply to other GM crops than soybean that would become considered “common allergens” in the future. Also, in addition to direct unintended effects of the GM on the intrinsic allergenicity of the whole GM plant, other kinds of unintended effects might be considered in the second generation of GM plants. An increase of allergenicity could result from interactions with the newly expressed proteins. In the

case of GM plants with a composition deliberately modified and/or with added nutritional/health value a possible increased consumption by particular at risk vulnerable groups of the population could also indirectly result in an unintended increase of the allergy risk. In such cases exposure assessment and post market monitoring programs may be recommended.

Towards rational assessment of changes in small molecules by leveraging genomics, metabolomics, and metabolic network modeling *(Seung Yon Rhee, Carnegie Institution for Science)*

Metabolism is a complex phenomenon where thousands of individual reactions are catalyzed by enzymes and regulated at many levels ranging from transcriptional changes of the enzymes to allosteric regulation of the enzymes by metabolites. Metabolism is coordinated at many levels of organization ranging from intracellular compartments to tissues, organs, and whole organisms.

Rational and quantitative assessment of metabolic changes in response to genetic modification is an open question and in need of innovative solutions. Non-targeted metabolite profiling can detect thousands of compounds, but it is not easy to understand the significance of the changed metabolites in the biochemical and biological context of the organism. In order to derive biochemical explanations or hypotheses for the observed metabolite changes from non-targeted metabolomics studies, it is important to examine the changed metabolites in the context of the genome-scale metabolic network of the organism.

Much progress has been made in the last few decades to represent metabolism at a genome scale (Terzer et al. 2009). The advances in genome sequencing and emerging fields such as biocuration and bioinformatics enabled the representation of genome-scale metabolic network reconstructions for model organisms (Bassel et al. 2012).

Two types of plant metabolic networks have been developed: descriptive and predictive. Genome-wide descriptive metabolic networks have been predicted from metabolic pathway databases that have been curated from experimental data in the literature such as MetaCyc (Caspi et al. 2012), PlantCyc (Zhang et al. 2010), and KEGG (Kanehisa et al. 2012). These genome-

wide metabolic network representations are now available for several plant species such as *Arabidopsis thaliana* (Mueller et al. 2003; Zhang et al. 2010), *Populus trichocarpa* (Zhang et al. 2010), *Chlamydomonas reinhardtii* (May et al. 2009), *Medicago truncatula* (Urbanczyk-Wochniak and Sumner 2007), grasses (Youens-Clark et al. 2011), and shade plants (Bombarely et al. 2011).

We developed a computational pipeline to generate the genome-scale metabolic networks of plant species. The pipeline includes automated prediction of enzymes, reactions, and metabolic pathways from a genome sequence and is highly accurate, transparent, scalable and flexible (Chae et al. 2014). It also includes a semi-automated pipeline to validate the predicted pathways, which has cut down manual validation time from weeks to hours. Using the pipeline, we have generated and publicly released metabolic networks of 16 plant and 1 algal species including model organisms *C. reinhardtii*, *A. thaliana*, *Brachypodium distachyon*, *Physcomitrella patens*, *Selaginella moellendorffii*, and *Populus trichocarp*a, and crop plants such as soybean, maize, *Manihot esculenta* (cassava), *Vitis vinifera* (grape wine), *Panicum virgatum* (switchgrass), barley, *Setaria italica* (foxmillet), *Brassica rapa* (Chinese cabbage), *Oryza sativa* (rice), *Carica papaya* (papaya), and *Sorghum bicolor* (sorghum). Comprehensive and comparative analyses of the metabolic potential across plant species will enable a more thorough and systematic analysis of unexpected pathways in genomes.

Predictive metabolic modeling approaches can be broadly grouped into kinetic and stoichiometric modeling (Sweetlove et al. 2008). Kinetic modeling uses enzyme kinetics to numerically simulate and test metabolic fluxes and can explain mechanism of flux changes. However, the difficulty of determining in vivo enzyme kinetics has limited this modeling to a small number of pathways. The most widely adapted modeling approach used for genome-wide metabolism is constraints-based modeling that uses stoichiometric, thermodynamic, and flux capacity constraints to model the fluxes of metabolites (Thiele and Palsson 2010). This approach has been used to build predictive models of metabolism for Arabidopsis (Poolman et al. 2009; de Oliveira Dal'Molin et al. 2010), maize (Saha et al. 2011), and *C. reinhardtii* (Chang et al. 2011). Most of these models have not been validated extensively using flux measurements, though

advances in metabolic flux analysis using ^{13}C-labeling and metabolomics approaches hold promise (Schwender 2008; Sweetlove et al. 2008; Allen et al. 2009).

These predictive models have been applied in a variety of studies ranging from metabolic engineering, drug discovery, drug target discovery, identification of novel gene function, evolutionary processes, network behaviors, and interpretations of mutant phenotypes (Feist and Palsson 2008). The most common algorithm used in these studies has been flux balance analysis (FBA), which attempts to balance the stoichiometry of the metabolites within the metabolic network system with a goal (objective function) of maximal growth or maximal biomass accumulation. While prediction of fluxes using FBA matches well with experimental data (Burgard and Maranas 2003), its assumptions may not always hold true, especially for engineered mutant lines. Several algorithms that have the goal of minimizing the change in the metabolic network upon perturbation have been developed, and appear to perform better than FBA in explaining fluxes of mutants (Segrè et al. 2002; Shlomi et al. 2005; Herrgård et al. 2006). This type of modeling could point to biochemical explanations for unintended or unexpected metabolite changes, which could help devise hypothesis-driven assessment strategies.

Using the genome-scale metabolic network of *A. thaliana*, we tested the effect of single genetic perturbations of 136 genes (129 knock-out and 7 overexpression lines) by comprehensively profiling the metabolites using 11 analytic platforms including GC-MS, LC-MS, and ESI (Quanbeck et al. 2012). Comparison of the metabolite profiles across the mutants showed that metabolic networks were robust to perturbations of single metabolic genes and the genetic perturbations changed the network more locally than globally (Kim and Rhee, unpublished results). This study revealed relationships between characteristics of the perturbed genes and metabolic changes. More analyses of this type would help in identifying the relationships between changed metabolites and their potential impact on the metabolic system and biology of the organism, which in turn would inform if altered composition could have toxic or other harmful effects for food and feed safety in any given crop.

Emerging tools and resources such as genome-scale metabolic networks, quantitative network modeling, and metabolomics may help assess the effects of genetic modification on metabolism

and may facilitate rational assessment of unintended effects of genetic modification on metabolism.

Food and feed safety of new plant varieties: how to assess unintended changes? *(Esther Kok, RIKILT Wageningen UR)*

There is global consensus on the basic approach for the assessment of unintended side effects of a genetic modification (Kleter et al. 2001; FAO/WHO 2004). This assessment will include in all cases a phenotypic and agronomic comparison between the new plant variety and a near comparator that is already on the market and considered as safe. In addition to this, for GM plant varieties a molecular characterization and a compositional analysis will form part of the safety assessment. From a scientific point of view, for current GM varieties this will usually be considered sufficient to conclude on potentially present unintended side effects of the genetic modification (Kok et al. 2008). Animal feeding trials with whole foods will in general be too insensitive to provide relevant information with relation to the safety of the new plant variety (Kuiper et al. 2013).

The discussion on potentially present unintended effects is nowadays much related to GM organisms. It is, however, clear in scientific literature that unintended side effects may occur in any type of breeding programme, including conventional crossing (Cellini et al. 2004; Kok et al. 2008). Moreover, new plant breeding programmes show a trend to the use of a broader range of molecular biological breeding techniques to achieve more complex genetic alterations within the framework of shorter breeding programs (Lusser et al. 2011). In the future, other strategies that aim for even more profound changes, such as strategies based on synthetic biology, may be applied to plant breeding. At the same time, it is observed that in general very little detailed information is available on current plant breeding programs, in scientific literature or any other publicly available source, and that so far food safety has not been a ‘standard’ item in plant breeding programs. As a result, there is limited basic data available for the assessment of changes in the physiology of plants that may affect the food safety of the plants and derived products.

Because of these combined developments, it may become increasingly interesting to have more informative analytical methods available that can screen the plant's physiology for unintended, potentially adverse, effects of the plant breeding program. At the same time, it is important to make sure that the assessment of unintended effects should be performed in the most cost-efficient manner, in order to prevent additional barriers for plant breeders. Omics technologies seem to meet both criteria. Omics technologies, whether transcriptomics, proteomics or metabolomics, have shown their added value already in different areas (Tanaka 2010; de Ligt et al. 2012; Rauch et al. 2012). In plant materials it has been shown that the methodologies can be applied in a reproducible and informative way (Fernie and Schauer 2009; Van Dijk et al. 2010, 2012; Oms-Oliu et al. 2013), confirming in a number of studies that differences between a single-trait transgenic plant variety and its conventional counterpart will in general be smaller than in the case of comparable conventional crosses, as can be expected.

Omics technologies can provide a detailed insight in potentially relevant changes in the physiology of the plant. In a first step, new plant varieties can be screened for any aberrant 'omics' profile, i.e., a profile that is different from the profiles of plant varieties that we consider as safe. This would not mean that the particular plant variety is not safe, but in the case of an aberrant profile a further detailed analysis of the new plant variety would be required to confirm the safety of the new plant variety. Similarly, new plant varieties for which the profiles fall within the band width of profiles from plant varieties that we consider as safe would not require further assessment. This approach links up to current approaches for targeted compositional analyses, but the information content in the case of omics technologies will be many-fold. Statistical and chemometric methods to rapidly screen profiles of new plant varieties with reference to profiles from plant varieties that we consider as safe are available (Van Dijk et al. 2014). In order to effectively implement omics technologies to improve current risk assessment procedures, there is a need to establish simple harmonized protocols for omics analyses and related data analyses with the aim to (i) compare the new plant variety to a near comparator and (ii) compare the new plant variety to a larger set of varieties of the same species that we consider as safe. This approach will in many cases link up to information that the plant breeder will already have available, thus considerably reducing the regulatory burden for plant breeders while at the same time safeguarding the food supply also in the years to come.

<u>Problem formulation in environmental risk assessment for GM crops</u> *(Alan Gray, CEH)*

Environmental risk assessment (ERA) for GM crops deals almost exclusively with the phenotype and therefore considers all plant traits that may have been altered by the transformation. These traits include both the intended effects of transformation (i.e., the effects of the inserted gene(s)) and any 'unintended' effects. Consideration of potential environmental risks of cultivating any particular GM crop usually follows a period of product development during which the novel crop is grown in multi-site trials alongside isogenic or near-isogenic lines. This long process of plant characterization exposes any meaningful and significant differences between the GM crop and its comparator, apart from those due to the insertion, by identifying any traits in the GM crop that display variation outside that of the normal range of the comparator. Of particular interest are any unintended changes in traits that may affect the biology of the GM plant in a way that increases the possibility of it becoming more persistent or invasive in either agricultural or natural environments. These include changes in the properties of the seeds (such as development rates, number, release from the plant (shattering), dormancy, and germination rates) that are important in the 'regeneration niche' of the plant's establishment and spread, and in those traits that affect the plant's competitiveness (such as seedling vigor, plant height, growth rates, and resistance to pests and disease). Data from plant characterization trials are therefore critically important in testing certain risk hypotheses regarding the environmental impact of the GM crop.

The subject matter of ERA is the collateral effects of cultivating a particular GM crop on a large scale, focusing especially on those effects that may cause harm. Problem Formulation is a method used in ERA to identify potentially harmful effects and to devise a plan to analyze the risks of the harm being realized. It does so by initially recognizing that 'harm' is a concept that is difficult to define except in relation to the objectives of environmental protection. Therefore the first stage in problem formulation is to identify a set of protection goals derived from local, national or international policy. These may be broadly stated (e.g., the Cartagena Protocol) or more specific laws, statutes, or even guidelines, but collectively they enable a risk assessor to identify those aspects of the environment that must be protected. Protection goals therefore enable us to identify those entities in the environment that must not be harmed. These can sometimes be formally defined in terms of 'assessment endpoints', which are explicit

expressions of an environmental value to be protected together with its attributes. For example ‘insect pollinator abundance’ is an assessment endpoint that may conceivably be affected by the large-scale cultivation of a specific GM crop if there was reason to imagine that the crop might impact the populations of insect pollinators, e.g., because it expressed an insecticidal protein.

The second stage of problem formulation is to seek a link between the cultivation of the GM crop and the assessment endpoint that may result in harm. This search for ‘pathways to harm’, alternatively described as developing an exposure scenario, lies at the heart of the ERA and provides the basis for testing a series of risk hypotheses about the likelihood of harm being realized. For example, insect pollinators are likely to be harmed if the plant presents a hazard (an insecticidal protein that negatively affects the insect) to which the insect may be exposed (if it is expressed in a part of the plant, in this case pollen, that may be ingested by the insect). Exposure to the hazard involves a set of contingent events (expression in the pollen, ingestion by the insect) the possibility of which can be examined in a conceptual model. Steps along the pathway to harm can be recast as risk hypotheses that can be validated or rejected from existing data or by designing new experiments or trials where appropriate. Such risk hypotheses are expressed as negatives and their validation early in the pathway increases confidence in any ‘no harm’ verdict. For example, validation of the hypotheses ‘the insect is not harmed by the protein’ or ‘the protein is not expressed in pollen’ allows a confident assessment of risk without further experimentation. Wolt et al. (2010) describe the process in detail, and Raybould (2011) and earlier papers referred to therein give specific examples of formulating and testing risk hypotheses. Gray (2012) and Tepfer et al. (2013) give practical examples of the use of problem formulation in ERA for GM crops.

Whereas ERA for GM crops embraces any unintended effects of transformation, the large-scale cultivation of such crops (indeed any novel crops) may have outcomes, whether negative or beneficial, that are ‘unintended’ in the sense that it was not the grower’s deliberate intention that they should happen. Variable in space and time, such contingent effects are fundamentally different from unintended effects of genetic transformation that are intrinsic properties of a GM plant. Problem formulation is designed to capture the negative effects, but unintended environmental effects may equally have environmental benefits (e.g., the increase in no-till

cultivation in the Canadian canola crop from 16% in 1996 to 46% in 2006 as more than 90% of the area was planted with herbicide-tolerant cultivars).

Can unintended effects lead to increased weediness / invasiveness? *(Paul Keese, OGTR, Australia)*

Possibly. A common approach is to identify any phenotypic or genotypic changes that may be a result of genetic modification and then determine if they are significant: namely, do they fall outside the parameters of substantial equivalence. This can be daunting if we consider unintended effects in the broadest sense as any molecular or phenotypic change, predicted or unpredicted.

Alternatively, we can limit our search to only those characteristics that are relevant to weediness/invasiveness. In Australia, these characteristics have been based on practical experience with more 1200 major environmental and agricultural weeds in diverse landscapes, including alpine regions, wetlands, rainforests, savannas, and deserts. In response, weed scientists have produced a robust and simple weed risk assessment protocol that can be readily applied to any plant. In addition, the large datasets available from weed risk assessments include plants across the whole risk spectrum, and allow rigorous validation tests to be conducted (Stone and Byrne 2011; Virtue et al. 2008).

The most advanced method for weed risk assessment is based on the post-border weed risk management protocol (Auld 2012), which was developed as a means of prioritizing existing weeds for control. It can be adapted to risk assessment of GM plants (Keese et al. 2014) by comparing the GM plant (including intended and unintended effects) to the weed risk of the parental species. This approach is used to identify significant changes based on three factors: the risk context, ability of the GM plant to spread and persist, and potential negative impacts. Establishing the risk assessment context takes into account the receiving environment, which is determined by those locations where the GM plant is predicted to be present, either through deliberate release of plant propagules or through spread of GM plants. The risk is calculated for each relevant land use (receiving environment). The management objective of a land use

(ecosystem services) may be primary production (e.g., agriculture, forestry), conservation (e.g., nature reserve), or human services (e.g., residential, water supply, roadsides). These types of environments may have varying susceptibility to invasion and different protection goals. Consequently, the risk score may differ between land uses.

The ability of the GM plant to spread and persist in the environment is a measure of its potential for invasiveness. The degree of invasiveness is determined by the following traits:

- ability to establish
  - ability of seedlings to survive amongst existing vegetation
  - tolerance of abiotic factors such as climate, soil type or rainfall patterns
  - tolerance of biotic factors such as pests and pathogens
  - tolerance of management control measures such as tilling, slashing or treatment with herbicides
- ability to reproduce
  - time to seed setting
  - number of seed set
  - ability to reproduce via vegetative propagules
- long-distance dispersal ability
  - by natural means such as birds, other animals, wind, or water
  - by human mediated means such as deliberate, accidental, via machinery, or via farm animals

The potential for giving rise to negative impacts considers traits that may:

- reduce biodiversity
- negatively affect the health of people or desired organisms
- reduce establishment of desired vegetation
- reduce yield or amount of desired vegetation
- reduce the quality of products or services
- restrict physical movement of people, animals, vehicles, machinery, and/or water
- negatively affect environmental health
  - provide food/shelter for pests and/or pathogens

- cause adverse fire regimes
- cause adverse soil nutrient levels, salinity, stability, water table levels

In conclusion, unintended effects may result in traits that are beneficial, adverse, or neutral. The risk assessment is typically restricted to consideration of adverse effects. However, most adverse effects arise from reduced plant performance that leads to undesirable agronomic traits. These plants are deliberately excluded during the screening process. Other unintended effects may be lost during backcrossing. Detection of any remaining changes that may give rise to adverse effects can be identified through recent advances in weed risk assessment methodology.

The post-border weed risk assessment approach provides for a systemic screening of relevant characteristics of GM plants that affect spread and persistence (invasiveness) and those that potentially give rise to negative impacts on human or animal health, or the environment. These characteristics capture changes due to either intended or unintended effects. Changes that have no or negligible effect on weed risk need not be explored. The post-border weed risk assessment approach therefore provides guidance on the data requirements, for both intended and unintended traits, that are considered relevant for ERA of a GM plant.

Nevertheless, the degree of change that is required to have a significant effect on increasing the weed risk score is unlikely to be achieved through unintended changes. This is consistent with experience from conventional breeding that has generated large numbers of new crop variants, but show scant evidence of increased weediness/invasiveness potential. Therefore, it is possible that unintended effects can lead to increased weediness/invasiveness, but it is unlikely.

Assessing the impact of unintended, transformation-related effects on non-target organisms

(Jörg Romeis, Agroscope, Institute for Sustainability Sciences ISS)

A common concern associated with the growing of GM crops is their potential to have adverse impacts on non-target organisms. Arthropods in particular form a major part of the biodiversity in agricultural landscapes and many are valued because they provide important ecosystem services, including biological control, pollination and decomposition, or cultural services

(Sanvido et al. 2012; Garcia-Alonso and Raybould 2014). Therefore, potential impacts that GM plants may have on non-target arthropods (NTAs) are addressed in ERA.

In the problem formulation phase, conceptual models are formulated that delineate how the cultivation of a GM crop could cause harm to valued non-target organisms, i.e., the entities to be protected (Gray 2012). This allows one to develop risk hypotheses that can be tested in the analytical phase of the risk assessment. The ERA follows a comparative approach, i.e., it focuses on those characteristics of the GM crop that differ from the non-transformed counterpart. The GM crop can differ from its non-transformed counterpart in two ways. First, it will produce one or several new molecules (e.g., a Cry toxin) that are responsible for the novel trait (e.g., the resistance against a specific set of insect pests). Second, the genetic modification of the plant may introduce unintended changes in respect to the crop composition or phenotype. Both the intended change (e.g., the production of a Cry toxin) as well as the unintended changes could cause unintended effects to valued non-target organisms. Since consequences of the intended change can be anticipated, it is possible to construct pathways (conceptual models) how growing of the GM plant could harm valued NTAs and to formulate testable risk hypotheses. A common hypothesis is that the stressor (i.e., the Cry toxin) does not reduce the abundance and ecological functions of NTAs under field conditions. This hypothesis is typically tested within a tiered framework that moves from laboratory or early-tier tests, using test species that are available and amenable to testing and have a high ability to detect potential hazards, to more complex (higher-tier) experiments that evaluate the risks under more realistic exposure conditions, including field studies (Romeis et al. 2008). Laboratory studies (termed tier 1 tests) are particularly powerful to test the risk hypothesis and in case that no adverse effects are detected under these highly controlled laboratory and worst-case exposure conditions, a “no effect” conclusion can be drawn with high confidence (Romeis et al. 2011).

In the case of unintended, plant-transformation-related effects, the assessment typically follows a weight-of-evidence approach taking into account information from the molecular characterization of the particular GM event and from comparisons of composition and agronomic and phenotypic characteristics of the GM plant with its non-GM counterpart (Garcia-Alonso and Raybould 2014). In the case that differences are detected, their likely biological relevance will be

assessed taking into account the range of values known for the conventional crop varieties that have a history of safe use. The aim of this assessment is to identify potentially harmful unintended changes that would then trigger a more detailed assessment (Romeis et al. 2008). This approach is considered sufficiently conservative given the fact that more than 99% of all transformation events are eliminated during prior agronomic and phenotypic analyses (e.g., Phillips McDougall 2011). Thus plants that show unusual or strange phenotypes are removed from the further breeding process.

Nevertheless, it is sometimes argued that risk assessments should include experiments to study the impact of unintended, transformation-related effects on non-target organisms. For example, EFSA requests non-target studies using GM plant material as a test substance to "… give indications on possible interactions between plant compounds and reflect realistic exposure conditions through bioavailability" (EFSA 2010). The justification for these additional data is that the compositional analyses do not necessarily target specific metabolites known to be involved in NTO–plant relationships. This approach, however, has various limitations. For example, it is usually unknown which metabolites are involved in these interactions and different metabolites are likely to affect different non-target species differently. Thus the stressor of concern is not well defined, i.e., it is the GM plant itself. Consequently, one needs to compare tissue from a GM plant with that from its closest non-GM comparator, ideally the near-isogenic line. This, however, poses a problem since the GM and non-GM lines will always differ in more than the transgene, which is a consequence of the breeding process that follows the genetic modification (Herman and Price 2013). With the increasingly developed stacked events that are created by conventional breeding the situation becomes more complex because a non-transformed near-isoline for direct comparison does not exist. Consequently, it is to be expected that experiments using GM plant material and conventional counterparts that reveal differences are difficult (or impossible) to interpret, in particular since a baseline for comparison is lacking. Such experiments and their results may add confusion rather than certainty to the ERA. The published literature on the non-target impact of Bt maize, for example, provides a number of examples where studies using GM plant tissue as test substance have resulted in inconclusive results (Romeis et al. 2013).

As a solution, unintended, transformation-related effects that have the potential to adversely affect NTAs need to be identified in the problem formulation phase of the ERA taking into account the results from the molecular, phenotypic, and agronomic characterization and the compositional analyses. If such characteristics are identified as stressors of concern, pathways to harm can be constructed and testable risk hypotheses can be formulated. This is a precondition to design and execute meaningful studies that provide data to support the ERA.

REFERENCES

Allen DK, Libourel IG, Shachar-Hill Y (2009) Metabolic flux analysis in plants: coping with complexity. Plant Cell Environ 32:1241–1257

Altman A, Hasegawa PM (2011) Plant biotechnology and agriculture: Prospects for the 21st century. Academic Press (Elsevier), London

Ames BN, Gold LS (2000) Paracelsus to parascience: the environmental cancer distraction. Mutation Res 447:3–13

Auld B (2012) An overview of pre-border weed risk assessment and post-border weed risk management protocols. Plant Prot Q 7:105–111

Bassel GW, Gaudinier A, Brady SM, Hennig L, Rhee SY, Smet ID (2012) Systems analysis of plant functional, transcriptional, physical interaction, and metabolic networks. Plant Cell 24:3859–3875

Bolhaar ST, van de Weg WE, van Ree R, Gonzalez-Mancebo E, Zuidmeer L, Bruijnzeel-Koomen CA, Fernandez-Rivas M, Jansen J, Hoffmann-Sommergruber K, Knulst AC, Gilissen LJ (2005) In vivo assessment with prick-to-prick testing and double-blind, placebo-controlled food challenge of allergenicity of apple cultivars. J Allergy Clin Immunol 116:1080–1086

Bombarely A, Menda N, Tecle IY, Buels RM, Strickler S, Fischer-York T, Pujar A, Leto J, Gosselin J, Mueller LA (2011) The Sol Genomics Network (solgenomics.net): growing tomatoes using Perl. Nucleic Acids Res 39(Database issue):D1149–1155

Bourgard, F, Gravot, A, Milesi, S, Gontier, E (2001) Production of plant secondary metabolites: a historical perspective. Plant Sci 161:839–851

Bregitzer P, Brown RH (2013) Long-term assessment of transgene behavior in barley: *Ds*-mediated delivery of *bar* results in robust, stable, and heritable expression. In Vitro Cell Dev Biol Plant 49:231–239

Bregitzer P, Dahleen LS (2008) A single backcross effectively eliminates agronomic and quality alterations caused by somaclonal variation in barley. Crop Sci 48:471–479

Bregitzer P, Halbert SE, Lemaux PG (1998) Somaclonal variation in the progeny of transgenic barley. Theor Appl Genet 96:421–425

Burgard AP, Maranas CD 2003 Optimization-based framework for inferring and testing hypothesized metabolic objective functions. Biotechnol Bioeng 82:670–677

Caspi R, Altman T, Dreher K, Fulcher CA, Subhraveti P, Keseler IM, Kothari A, Krummenacker M, Latendresse M, Mueller LA, Ong Q, Paley S, Pujar A, Shearer AG, Travers M, Weerasinghe D, Zhang P, Karp PD (2012) The MetaCyc database of metabolic pathways and enzymes and the BioCyc collection of pathway/genome databases. Nucleic Acids Res 40(Database issue):D742–753

Castiglioni P, Warner D, Bensen RJ, Anstrom DC, Harrison J, Stoecker M, Abad M, Kumar G, Salvador S, D'Ordine R, Navarro S, Back S, Fernandes M, Targolli J, Dasgupta S, Bonin C, Luethy MH, Heard JE (2008) Bacterial RNA chaperones confer abiotic stress tolerance in plants and improved grain yield in maize under water-limited conditions. Plant Physiol 147:446–455

Cellini F, Chesson A, Colquhoun I, Constable A, Davies HV, Engel KH, Gatehouse AM, Kärenlampi S, Kok EJ, Leguay JJ, Lehesranta S, Noteborn HP, Pedersen J, Smith M (2004) Unintended effects and their detection in genetically modified crops. Food Chem Toxicol 42:1089–1125

Chae L, Kim T, Nilo-Poyanco R, Rhee SY (2014) Genomic signatures of specialized metabolism in plants. Science 344:510–513

Chaikam V, Karlson D (2008) Functional characterization of two cold shock domain proteins from *Oryza sativa*. Plant Cell Environ 31: 995–1006

Chang RL, Ghamsari L, Manichaikul A, Hom EF, Balaji S, Fu W, Shen Y, Hao T, Palsson BØ, Salehi-Ashtiani K, Papin JA (2011) Metabolic network reconstruction of *Chlamydomonas* offers insight into light-driven algal metabolism. Mol Syst Biol 7:518

Chen CH, Dickman KG, Moriya M, Zavadil J, Sidorenko VS, Edwards KL, Gnatenko DV, Wu L, Turesky RJ, Wu XR, Pu YS, Grollman AP (2012) Aristolochic acid-associated urothelial cancer in Taiwan. Proc Natl Acad Sci USA 109:8241–8246

Chen H, Lin Y (2013) Promise and issues of genetically modified crops. Curr Opin Plant Biol 16:255–260

Codex Alimentarius (2003) Guideline for the Conduct of Food Safety Assessment of Foods Derived from Recombinant-DNA Plants. CAC/GL 45-2003

Craig W, Tepfer M, Degrassi G, Ripandelli D (2008) An overview of general features of risk assessments of genetically modified crops. Euphytica 164:853–860

Cristofari G, Darlix JL (2002) The ubiquitous nature of RNA chaperone proteins. Prog Nucleic Acid Res Mol Biol 72:223–268

de Ligt J, Willemsen MH, van Bon BW, Kleefstra T, Yntema HG, Kroes T, Vulto-van Silfhout AT, Koolen DA, de Vries P, Gilissen C, del Rosario M, Hoischen A, Scheffer H, de Vries BB, Brunner HG, Veltman JA, Vissers LE (2012) Diagnostic exome sequencing in persons with severe intellectual disability. N Engl J Med 367:1921–1929

de Oliveira Dal'Molin CG, Quek LE, Palfreyman RW, Brumbley SM, Nielsen LK (2010) AraGEM, a genome-scale reconstruction of the primary metabolic network in Arabidopsis. Plant Physiol 152:579–589

EC (2013) Commission Implementing Regulation (EU) No 503/2013 of 3 April 2013 on applications for authorisation of genetically modified food and feed in accordance with Regulation (EC) No 1829/2003 of the European Parliament and of the Council and amending Commission Regulations (EC) No 641/2004 and (EC) No 1981/2006. Official Journal of the European Union, L157 pp 1–48

EFSA (2010) Guidance on the environmental risk assessment of genetically modified plants. EFSA J 8:1879

EFSA (2011) EFSA Panel on Genetically Modified Organisms (GMO). Guidance for risk assessment of food and feed from genetically modified plants. EFSA J 9:2150

Evans J, Wood G, Miller M (2006) The risk assessment – policy gap: an example from the UK contaminated land regime. Environ Int 32: 1066–1071

FAO/WHO (2004) Foods derived from biotechnology. Joint FAO/WHO Food Standards Programme, Codex Alimentarius Commission

Fedoroff NV (1989) About maize transposable elements and development. Cell 56:181–191

Feist AM, Palsson BØ (2008) The growing scope of applications of genome-scale metabolic reconstructions using *Escherichia coli*. Nat Biotechnol 26:659–667

Fernandez A, Mills ENC, Lovik M, Spoek A, Germini A, Mikalsen A, Wal JM (2013) Endogenous allergens and compositional analysis in the allergenicity assessment of genetically modified plants. Food Chem Toxicol 62:1–6

Fernie AR, Schauer N (2009) Metabolomics-assisted breeding: a viable option for crop improvement? Trends Genet 25:39–48

Foetisch K, Dahl L, Jansen B, Becker WM, Lidholm J, van Ree R, Broll H, Kaul S, Vieths S, Holzhauser T (2011) Development and in-house validation of allergen-specific ELISA tests for the quantification of Dau c 1.01, Dau c 1.02 and Dau c 4 in carrot extracts (*Daucus carota*). Anal Bioanal Chem 399:935–943

Forsbach A, Schubert D, Lechtenberg B, Gils M, Schmidt R (2003) A comprehensive characterization of single-copy T-DNA insertions in the *Arabidopsis thaliana* genome. Plant Mol Biol 52:161–176

Gao Z, van de Weg EW, Matos CI, Arens P, Bolhaar ST, Knulst AC, Li Y, Hoffmann-Sommergruber K, Gilissen LJ (2008) Assessment of allelic diversity in intron-containing Mal d 1 genes and their association to apple allergenicity. BMC Plant Biol 8:116

Garcia-Alonso M, Raybould A (2014) Protection goals in environmental risk assessment: a practical approach. Transgenic Res 23:945–956

Goodman RE, Panda R, Ariyarathna H (2013) Evaluation of endogenous allergens for the safety evaluation of genetically engineered food crops: review of potential risks, test methods, examples and relevance. J Agric Food Chem 61:8317–8332

Gray A (2012) Problem formulation in environmental risk assessment for genetically modified crops: a practitioner's approach. Colln Biosafety Rev 6:10–65

Hayder H, Mueller U, Bartholomaeus A (2011) Review of intolerance reactions to food and food additives. Int Food Risk Anal J 1:23–32

Herman RA, Price WD (2013) Unintended compositional changes in genetically modified (GM) crops: 20 years of research. J Agric Food Chem 61:11695–11701

Herrgård MJ, Fong SS, Palsson BØ (2006) Identification of genome-scale metabolic network models using experimentally measured flux profiles. PLoS Comput Biol 2(7):e72

Kanehisa M, Goto S, Sato Y, Furumichi M, Tanabe M (2012) KEGG for integration and interpretation of large-scale molecular data sets. Nucleic Acids Res 40(Database issue):D109–114

Keese PK, Robold AV, Myers RC, Weisman S, Smith J (2014) Applying a weed risk assessment approach to GM crops. Transgenic Res 23:957–969

Kessler DA, Taylor MR, Maryanski JH, Flamm EL, Kahl LS (1992) The safety of foods developed by biotechnology. Science 256:1747–1749

Kleter GA, van der Krieken WM, Kok EJ, Bosch D, Jordi W, Gilissen JWJ (2001) Regulation and exploitation of genetically modified crops. Nat Biotechnol 19:1105–1110

Kok EJ, Keijer J, Kleter GA, Kuiper HA (2008) Comparative safety assessment of plant-derived foods. Regul Toxicol Pharmacol 50:98–113

Krattinger SG, Lagudah ES, Spielmeyer W, Singh RP, Huerta-Espino J, McFadden H, Bossolini E, Selter LL, Keller B (2009) A putative ABC transporter confers durable resistance to multiple fungal pathogens in wheat. Science 323:1360–1363

Kuiper HA, Kok EJ, Davies HV (2013) New EU legislation for risk assessment of GM food: no scientific justification for mandatory animal feeding trials. Plant Biotechnol J 11:781–784

Larkin PJ, Scowcroft WR (1981) Somaclonal variation: A novel source of variability from cell cultures for plant improvement. Theor Appl Genet 60:197–214

Li J, Zhang Z (2013) miRNA regulatory variation in human evolution. Trends Genet 29:116–124

Lusser M, Parisi C, Plan D, Rodríguez-Cerezo E (2011) New plant breeding techniques: State-of-the-art and prospects for commercial development. IPTS report, JRC 63971, EUR 24760 EN. doi:10.2791/54761. Available from http://ftp.jrc.es/EURdoc/JRC63971.pdf. Accessed 20 October 2014

Ma J, Bennetzen JL (2004) Rapid recent growth and divergence of rice nuclear genomes. Proc Natl Acad Sci USA 101:12404–12410

Martineau B (2001) First fruit: The creation of the Flavr Savr tomato and the birth of biotech foods. McGraw-Hill, New York

May P, Christian JO, Kempa S, Walther D (2009) ChlamyCyc: an integrative systems biology database and web-portal for *Chlamydomonas reinhardtii*. BMC Genomics 10:209

McClintock B (1984) The significance of responses of the genome to challenge. Science 226: 792–801

Metcalfe DD, Astwood JD, Townsend R, Sampson HA, Taylor SL, Fuchs RL (1996) Assessment of the allergenic potential of foods derived from genetically engineered crop plants. Crit Rev Food Sci Nutr 36:S165–86

Moneret-Vautrin DA, Peltre G, Gayraud J, Morisset M, Renaudin JM, Martin A (2012) Prevalence of sensitisation to oilseed rape and maize pollens in France: a multi-center study carried out by the Allergo-Vigilance Network. Eur Ann Allergy Clin Immunol 44:225–235

Mueller LA, Zhang P, Rhee SY (2003) AraCyc: a biochemical pathway database for Arabidopsis. Plant Physiol 132:453–460

Nemali KS, Bonin C, Dohleman FG, Stephens M, Reeves WR, Nelson DE, Whitsel JE, Sammons B, Silady RA, Anstrom D, Sharp RE, Patharkar OR, Clay D, Coffin M, Nemeth MA,

Leibman ME, Luethy M, Lawson M (2014) Physiological responses related to increased grain yield under drought in the first biotechnology-derived drought tolerant maize. Plant Cell Environ doi: 10.1111/pce.12446 (epub ahead of print)

Nordlee JA, Taylor SL, Townsend JA, Thomas LA, Bush RK (1996) Identification of Brazil-nut allergen in transgenic soybeans. N Engl J Med 334:688–692

OECD (1993) Safety evaluation of foods derived by modern biotechnology: Concepts and principles. OECD, Paris

Oliver KR, JA McComb, and WK Greene (2013) Transposable elements: powerful contributors to angiosperm evolution and diversity. Genome Biol Evol 5:1886–1901

Oms-Oliu G, Odriozola-Serrano I, Martín-Belloso O (2013) Metabolomics for assessing safety and quality of plant-derived food. Food Res Int 54:1172–1183

Ossowski S, Schneeberger K, Lucas-Lledó JI, Warthmann N, Clark RM, Shaw RG, Weigel D, Lynch M (2010) The rate and molecular spectrum of spontaneous mutations in *Arabidopsis thaliana*. Science 327:92–94

Pagliarani G, Paris R, Arens P, Tartarini S, Ricci G, Smulders MM, van de Weg WE (2013) A qRT-PCR assay for the expression of all Mal d 1 isoallergen genes. BMC Plant Biol 13:51

Perlak FJ, Stone TB, Muskopf YM, Petersen LJ, Parker GB, McPherson SA, Wyman J, Love S, Reed G, Biever D, Fischhoff DA (1993) Genetically improved potatoes: protection from damage by Colorado potato beetles. Plant Mol Biol 22:313–321

Phillips McDougall (2011) The cost and time involved in the discovery, development and authorisation of a new plant biotechnology derived trait. http://croplife.org/plant-biotechnology/regulatory-2/cost-of-bringing-a-biotech-crop-to-market/. Accessed 14 Aug 2014

Poolman MG, Miguet L, Sweetlove LJ, Fell DA (2009) A genome-scale metabolic model of Arabidopsis and some of its properties. Plant Physiol 151:1570–1581

Prescott VE, Campbell PM, Moore A, Mattes J, Rothenberg ME, Foster PS, Higgins TJV, Hogan SP (2005) Transgenic expression of bean alpha-amylase inhibitor in peas results in altered structure and immunogenicity. J Agric Food Chem 53:9023–9030

Privalle LS, Chen J, Clapper G, Hunst P, Spiegelhalter F, Zhong CX (2012) Development of an agricultural biotechnology crop product: testing from discovery to commercialization. J Agric Food Chem 60:10179–10187

Quanbeck, SM, Brachova L, Campbell AA, Guan X, Perera A, He K, Rhee SY, Preeti Bais P, Dickerson JA, Dixon P, Wohlgemuth G, Fiehn O, Barkan L, Lange I, Lange BM, Lee I, Cortes D, Salazar C, Shuman J, Shulaev V, Huhman DV, Sumner LW, Roth MR, Welti R, Ilarslan H, Wurtele ES, Nikolau BJ (2012) Metabolomics as a hypothesis-generating functional genomics tool for the annotation of *Arabidopsis thaliana* genes of “unknown function”. Frontiers Plant Sci 3:1–12

Rauch A, Wieczorek D, Graf E, Wieland T, Endele S, Schwarzmayr T, Albrecht B, Bartholdi D, Beygo J, Di Donato N, Dufke A, Cremer K, Hempel M, Horn D, Hoyer J, Joset P, Röpke A, Moog U, Riess A, Thiel CT, Tzschach A, Wiesener A, Wohlleber E, Zweier C, Ekici AB, Zink AM, Rump A, Meisinger C, Grallert H, Sticht H, Schenck A, Engels H, Rappold G, Schröck E, Wieacker P, Riess O, Meitinger T, Reis A, Strom TM (2012) Range of genetic mutations associated with severe non-syndromic sporadic intellectual disability: an exome sequencing study. Lancet 380:1674–1682

Raybould A (2011) The bucket and the searchlight: formulating and testing risk hypotheses about the weediness and invasiveness potential of transgenic crops. Environ Biosafety Res 9:123–133

Risk JM, Selter, LL, Chauhan H, Krattinger, SG, Kumlehn J, Hensel G, Viccars LA, Richardson TM, Buesing G, Troller A, Lagudah ES, Keller B (2013) The wheat *Lr34* gene provides resistance against multiple fungal pathogens in barley. Plant Biotechnol J 11:847–854

Romeis J, Bartsch D, Bigler F, Candolfi MP, Gielkens MMC, Hartley SE, Hellmich RL, Huesing JE, Jepson PC, Layton R, Quemada H, Raybould A, Rose RI, Schiemann J, Sears MK, Shelton AM, Sweet J, Vaituzis Z, Wolt JD (2008) Assessment of risk of insect-resistant transgenic crops to nontarget arthropods. Nat Biotechnol 26:203–208

Romeis J, Hellmich RL, Candolfi MP, Carstens K, De Schrijver A, Gatehouse AMR, Herman RA, Huesing JE, McLean MA, Raybould A, Shelton AM, Waggoner A (2011) Recommendations for the design of laboratory studies on non-target arthropods for risk assessment of genetically engineered plants. Transgenic Res 20:1–22

Romeis J, McLean MA, Shelton AM (2013) When bad science makes good headlines: Bt maize and regulatory bans. Nat Biotechnol 31:386–387

Ryan C, Smyth SJ (2012) Economic implications of low-level presence in a zero-tolerance European import market: The case of Canadian Triffid Flax. AgBioForum 115:21–30

Saha R, Suthers PF, Maranas CD (2011) *Zea mays i*RS1563: a comprehensive genome-scale metabolic reconstruction of maize metabolism. PLoS One 6(7):e21784

Sammons B, Whitsel J, Stork LG, Reeves W, Horak M (2014) Characterization of drought-tolerant maize MON 87460 for use in environmental risk assessment. Crop Sci 54:719–729

Sancho AI, Foxall R, Browne T, Dey R, Zuidmeer L, Marzban G, Waldron KW, van Ree R, Hoffmann-Sommergruber K, Laimer M, Mills EN (2006a) Effect of postharvest storage on the expression of the apple allergen Mal d 1. J Agric Food Chem 54:5917–5923

Sancho AI, Foxall R, Rigby NM, Browne T, Zuidmeer L, van Ree R, Waldron KW, Mills EN (2006b) Maturity and storage influence on the apple (*Malus domestica*) allergen Mal d 3, a nonspecific lipid transfer protein. J Agric Food Chem 54:5098–5104

Sanvido O, Romeis J, Gathmann A, Gielkens M, Raybould A, Bigler F (2012) Evaluating environmental risks of genetically modified crops – ecological harm criteria for regulatory decision-making. Environ Sci Policy 15:82–91

Sarevitz D (2004) How science makes environmental controversies worse. Environ Sci Policy 7: 385–403

Schwender J (2008) Metabolic flux analysis as a tool in metabolic engineering of plants. Curr Opin Biotechnol 19:131–137

Segrè D, Vitkup D, Church GM (2002) Analysis of optimality in natural and perturbed metabolic networks. Proc Natl Acad Sci USA 99:15112–15117

Shlomi T, Berkman O, Ruppin E (2005) Regulatory on/off minimization of metabolic flux changes after genetic perturbations. Proc Natl Acad Sci USA 102:7695–7700

Steiner HY, Halpin C, Jez JM, Kough J, Parrott W, Underhill L, Weber N, Hannah LC (2013) Evaluating the potential for adverse interactions within genetically engineered breeding stacks. Plant Physiol 161:1587–1594

Stone LM, Byrne M (2011) Comparing the outputs of five weed risk assessment models implemented in Australia: are there consistencies across models? Plant Prot Q 26:29–35

Sweetlove LJ, Fell D, Fernie AR (2008) Getting to grips with the plant metabolic network. Biochem J 409:27–41

Tanaka H (2010) Omics-based medicine and systems pathology: A new perspective for personalized and predictive medicine. Methods Inf Med 49:173–185

Tepfer M, Racovita M, Craig W (2013) Putting problem formulation at the forefront of GMO risk analysis. GM Crops Food 4:10–15

Terzer M, Maynard ND, Covert MW, Stelling J (2009) Genome-scale metabolic networks. Wiley Interdiscip Rev Syst Biol Med 1:285–297

Thiele I, Palsson BØ (2010) A protocol for generating a high-quality genome-scale metabolic reconstruction. Nat Protoc 5:93–121

Tollenaar M, Lee EA (2010) Strategies for enhancing grain yield in maize. In: Janick J (ed) Plant breeding reviews, vol 34. John Wiley & Sons, Hoboken, NJ

Urbanczyk-Wochniak E, Sumner LW (2007) MedicCyc: a biochemical pathway database for *Medicago truncatula*. Bioinformatics 23:1418–1423

van Dijk JP, Leifert C, Barros E, Kok EJ (2010). Gene expression profiling for food safety assessment: Examples in potato and maize. Regul Toxicol Pharmacol 58:S21–S25

van Dijk JP, Cankar K, Hendriksen PJM, Beenen HG, Zhu M, Scheffer S, Shepherd LVT, Stewart D, Davies HV, Leifert C, Wilkockson SJ, Gruden K, Kok EJ (2012) The identification and interpretation of differences in the transcriptomes of organically and conventionally grown potato tubers. J Agric Food Chem 60:2090−2101

van Dijk JP, de Mello CS, Voorhuijzen MM, Hutten RCB, Arisi ACM, Jansen JJ, Buydens LMC, van der Voet H, Kok EJ (2014) Safety assessment of plant varieties using transcriptomics profiling and a one-class classifier. Regul Toxicol Pharmacol 70:297–303

Vaughn JN, Bennetzen JL (2014) Natural insertions in rice commonly form tandem duplications indicative of patch-mediated double-strand break induction and repair. Proc Natl Acad Sci USA 111:6684–6689

Virtue JG, Spencer JE, Weiss JE, Reichard SE (2008) Australia's Botanic Gardens weed risk assessment procedure. Plant Prot Q 23:166–178

Watson JD, Baker TA, Bell SP, Gann A, Levine M, Losick R (2013) Molecular biology of the gene, 7th edn. Cold Spring Harbor Laboratory Press. ISBN 978-0-321-76243-6

Weber N, Halpin C, Hannah LC, Jez J, Kough J, Parrott W (2012) Crop genome plasticity and its relevance to food and feed safety of genetically engineered breeding stacks. Plant Physiol 160:1842–1853

Wink M, ed. (2010) Annual Plant Reviews, Volume 40: Biochemistry of Plant Secondary Metabolism, 2nd edn. Wiley-Blackwell, Oxford, UK

Wolt JD, Keese P, Raybould A, Fitzpatrick JW, Burachik M, Gray A, Olin SS, Schiemann J, Sears M, Wu F (2010) Problem formulation in the environmental risk assessment for genetically modified plants. Transgenic Res 19:425–436

Youens-Clark K, Buckler E, Casstevens T, Chen C, Declerck G, Derwent P, Dharmawardhana P, Jaiswal P, Kersey P, Karthikeyan AS, Lu J, McCouch SR, Ren L, Spooner W, Stein JC, Thomason J, Wei S, Ware D (2011) Gramene database in 2010: updates and extensions. Nucleic Acids Res 39(Database issue):D1085–1094

Zhang P, Dreher K, Karthikeyan A, Chi A, Pujar A, Caspi R, Karp P, Kirkup V, Latendresse M, Lee C, Mueller LA, Muller R, Rhee SY (2010) Creation of a genome-wide metabolic pathway database for *Populus trichocarpa* using a new approach for reconstruction and curation of metabolic pathways for plants. Plant Physiol 153:1479–1491

Zuidmeer L, Goldhahn K, Rona RJ, Gislason D, Madsen C, Summers C, Sodergren E, Dahlstrom J, Lindner T, Sigurdardottir ST, McBride D, Keil T (2008) The prevalence of plant food allergies: a systematic review. J Allergy Clin Immunol 121:1210–1218
